# Annealing‐Induced Plasticity and Strengthening in Metallic Glasses

**DOI:** 10.1002/advs.202524294

**Published:** 2026-02-23

**Authors:** Yingjie Zhang, Jinyue Wang, Leqing Liu, Yuan Wu, Yi‐huan Cao, Jiqi Yu, Zhen Tian, Zhichao Lu, Fengshou Li, Qiang Chen, Guosheng Zhang, Laiquan Shen, Yong Yu, Hui Wang, Suihe Jiang, Xiaobin Zhang, Xiongjun Liu, Zhaoping Lu

**Affiliations:** ^1^ State Key Laboratory for Advanced Metals and Materials University of Science and Technology Beijing Beijing China; ^2^ School of Intelligent Manufacturing and Mechanical Engineering Hunan Institute of Technology Hengyang China; ^3^ School of Mathematics and Physics University of Science and Technology Beijing Beijing China; ^4^ Institute for Materials Intelligent Technology Liaoning Academy of Materials Shenyang China; ^5^ Institute of Physics Chinese Academy of Sciences Beijing China; ^6^ Neutron Science Center Songshan Lake Materials Laboratory Dongguan China

**Keywords:** annealing toughening, chemical design, compensatory plasticity, metallic glasses, strength‐ductility synergy, structural heterogeneity

## Abstract

Annealing‐induced embrittlement has long been considered unavoidable in metallic glasses because it annihilates free volume (i.e., locally loosely‐packed regions, LLPRs) required for plasticity. Here, we overturn this paradigm by demonstrating that sub‐*T*_g_ annealing simultaneously increases both the strength and compressive plasticity of a chemically tailored Zr‐based metallic glass, with plastic strain increasing by >150%. Our experimental analyses reveal that strategically employed Ni─Cu repulsion drives elemental partitioning during annealing, which seeds nanoscale chemical heterogeneity. Concurrently, atomistic simulations suggest the emergence of locally densely‐packed regions (LDPRs) with characteristically low activation energy for shear transformation. These findings indicate that plasticity can be sustained by heterogeneous structures wherein densely‐packed motifs, alongside conventional loosely‐packed regions, serve as potential shear transformation sites. This shifts the design paradigm from merely introducing LLPRs to strategically engineering heterogeneous structures that enable compensatory plasticity.

## Graphical abstract

This work challenges the dogma that annealing inevitably embrittles metallic glasses. We demonstrate that sub‐*T*_g_ annealing, guided by positive mixing enthalpy and constrained kinetics, can drive a self‐organized compositional fluctuation that creates an interface‐free heterogeneous structure. Within this structure, deformable densely‐packed regions (LDPRs) arise to compensate for the vanishing loose‐packed regions (LLPRs), enabling a simultaneous increase in strength and ductility exceeding 150% through compensatory plasticity.

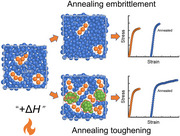

## 1 Introduction

Bulk metallic glasses (BMGs) are renowned for their exceptional strength and elastic limit, a direct consequence of their disordered atomic architecture [1, 2]. Yet, this intrinsic high strength is invariably shadowed by a catastrophic macroscopic brittleness, because plastic strain localizes into nanometer‐thin shear bands instead of distributing homogeneously [3, 4, 5]. To circumvent this limitation, extensive research has pursued microstructural engineering through ultra‐rapid quenching [6, 7, 8], pre‐deformation [4, 9, 10], irradiation [11, 12, 13], or thermal cycling [14, 15, 16]. A unifying principle underpins these approaches: the deliberate introduction of excess free volume, which manifests as locally loosely packed regions (LLPRs)—structurally frustrated motifs that serve as preferential nucleation sites for shear transformation zones (STZs), the fundamental carriers of plasticity [14, 17, 18, 19].

Unfortunately, the thermodynamic drive toward equilibrium inherently undermines this LLPR‐based design principle. Structural relaxation, even below the glass transition temperature (*T*_g_), progressively annihilates the quenched‐in excess volume: LLPRs disappear, and the glass densifies into lower‐enthalpy configurations [19, 20, 21, 22, 23]. This evolution enhances yield strength but precipitously diminishes plasticity and toughness, a trade‐off starkly documented in benchmark BMGs such as Vitreloy‐1 [22] and its counterparts. For decades, reconciling this annealing‐induced strengthening with a catastrophic loss of toughness has represented a fundamental dilemma in glass physics, cementing the paradigm that any thermal relaxation inevitably embrittles MGs.

Here, we report a decisive break from the “annealing‐equals‐embrittlement” paradigm. In a strategically designed Zr‐based BMG, sub‐*T*_g_ annealing simultaneously enhances both strength and compressive plasticity by more than 150%. This counterintuitive behaviour is enabled by a chemical design that leverages a positive heat of mixing to transform the relaxation pathway. We show that annealing triggers an enthalpy‐driven atomic reorganization, which reduces LLPRs (i.e., excess free volume) while seeding a high density of locally densely packed regions (LDPRs). Atomistic simulations reveal a dual activation‐carrier mechanism that validates the early theoretical insight that high‐energy, crowded atomic environments can initiate plasticity [24, 25]. Crucially, we demonstrate that STZs nucleate preferentially at high‐energy sites distributed across both LLPRs and LDPRs, with the annealing‐induced LDPRs exhibiting characteristically low activation barriers for shear.

As a result, the proliferation of deformable LDPRs (i.e., densification of the glass) supplies new readily activated plasticity carriers that compensate for the loss of LLPRs and turn annealing into a toughening strategy. Our work demonstrates that plasticity is governed not by the number of LLPRs alone, but by the energy landscape of accessible atomic configurations whose activation barriers dictate shear initiation. These findings redefine design principles for damage‐tolerant glasses, opening a route to simultaneously high strength and large ductility in MGs.

## 2 Results

### 2.1 Annealing‐Induced Simultaneous Strengthening and Toughening

**FIGURE 1:**
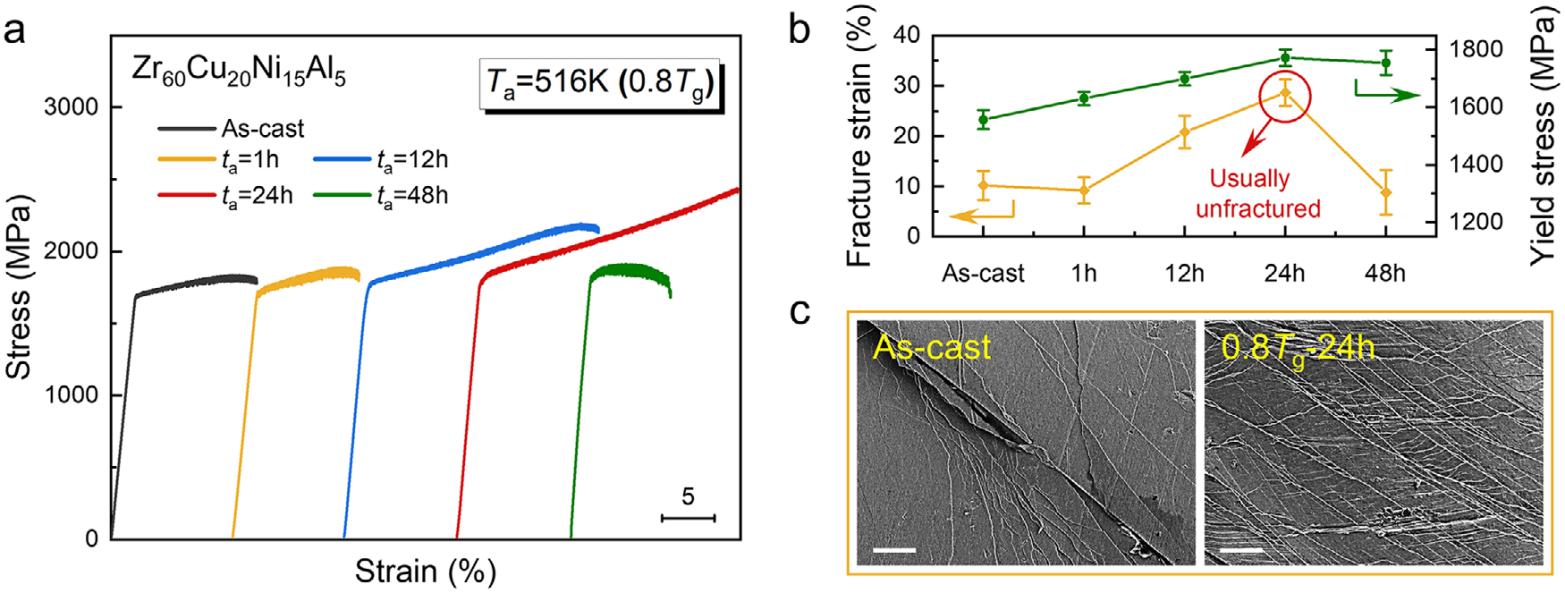
Anomalous strength–ductility synergy in Ni15 BMG annealed at 0.8 *T*_g_. (a) Compressive engineering stress–strain curves revealing the effect of sub‐*T*_g_ annealing on mechanical properties. (b) Fracture strength (*σ*_f_) and plastic strain (*ε*_p_) vs. annealing time, both peaking at 24 h. (c) SEM side views of fractured samples: the as‐cast exhibits sparse shear bands while the 24 h annealed displays dense, numerous shear bands. Scale bars: 50 µm.

The pursuit of ductile BMGs has long been guided by a fundamental constraint: sub‐*T*_g_ annealing invariably triggers embrittlement. We overturned this rule in the Zr‐based Zr_60_Cu_20_Ni_15_Al_5_ BMG (hereinafter referred to as Ni15), and Figure 1 shows its mechanical response after isothermal sub‐*T*_g_ annealing treatments. During isothermal annealing at 0.80 *T*_g_ (516 K), the compressive yield strength rises continuously from 1557 ± 33 to 1772 ± 29 MPa while the plastic strain more than doubles from ∼10% to >25%, as the annealing duration was prolonged from 1 to 24 h (Figure 1a,b). Concurrently, the Young's modulus exhibits a consistent increase (Table S1). After longer exposures (e.g., 48 h), the strength remains high (1754 ± 42 MPa) but plastic strain falls back to ∼10%, revealing an optimal time window. The same trend was observed at other sub‐*T*_g_ annealing temperatures, such as 0.85 *T*_g_ (548 K) and 0.90 *T*_g_ (580 K) (Figure S1).

This anomalous toughening is physically manifested by the deformation morphology. Scanning electron microscopy (SEM) observation reveals a dramatic transformation in shear banding behavior after 24 h of annealing (Figure 1c). The shear band spacing narrows from ∼60 µm in the as‐cast state to ∼7 µm, concomitant with a surge in number density from ∼0.02 to ∼0.14 µm^−1^. This profuse branching pattern signifies a fundamental shift from unstable, highly localized plasticity to a mode with a vastly increased number of easily activated shear sites that can sustain homogeneous deformation.

This unexpected toughening is intrinsic to the designed atomic structure of Ni15. Under identical annealing conditions, the conventional Zr_60_Cu_30_Al_10_ (Ni0) BMG followed the classic embrittlement trajectory, with compressive plasticity diminishing after just 6 h annealing and culminating in brittle fracture within 24 h (Figure S2). This stark mechanical bifurcation unambiguously confirms that annealing‐induced ductilization is a unique property of alloy Ni15, which is contrary to the wisdom of annealing‐induced embrittlement.

### 2.2 Emergent Structural Heterogeneity

To reveal the underlying mechanism of the anomalous annealing‐induced ductilization, we first examined the structural characteristics of Ni15 after annealing. In contrast to the occurrence of nanoscale ordering in other annealed BMGs, high‐resolution transmission electron microscopy (HRTEM) and selected‐area electron diffraction (SAED) verified that the alloy retains its fully amorphous state within the optimal annealing window (Figure S4), thereby excluding crystallization or precipitation as a plausible source for the observed toughening [26, 27, 28, 29, 30, 31, 32].

We next quantified the extent of structural relaxation. Differential scanning calorimetry (DSC) revealed a progressive suppression of the pre‐*T*_g_ exothermic peak (Figure S5, inset), signifying annihilation of excess free volume. The associated enthalpy release (∆*H*) drops by ∼80% after the 24‐h anneal (Figure S5, Note S1) and is well‐described by the Kohlrausch‐Williams‐Watts kinetic model [33], indicating that the glass has approached a relaxed configuration. The simultaneous increase in density (Figure S5) and Vickers hardness (Figure S6) confirms that excess free volume and its associated LLPRs have been largely eliminated. This definitive evidence of global relaxation raises the obvious question of what replaces the lost plasticity carriers and delivers the observed macroscopic plasticity.

**FIGURE 2:**
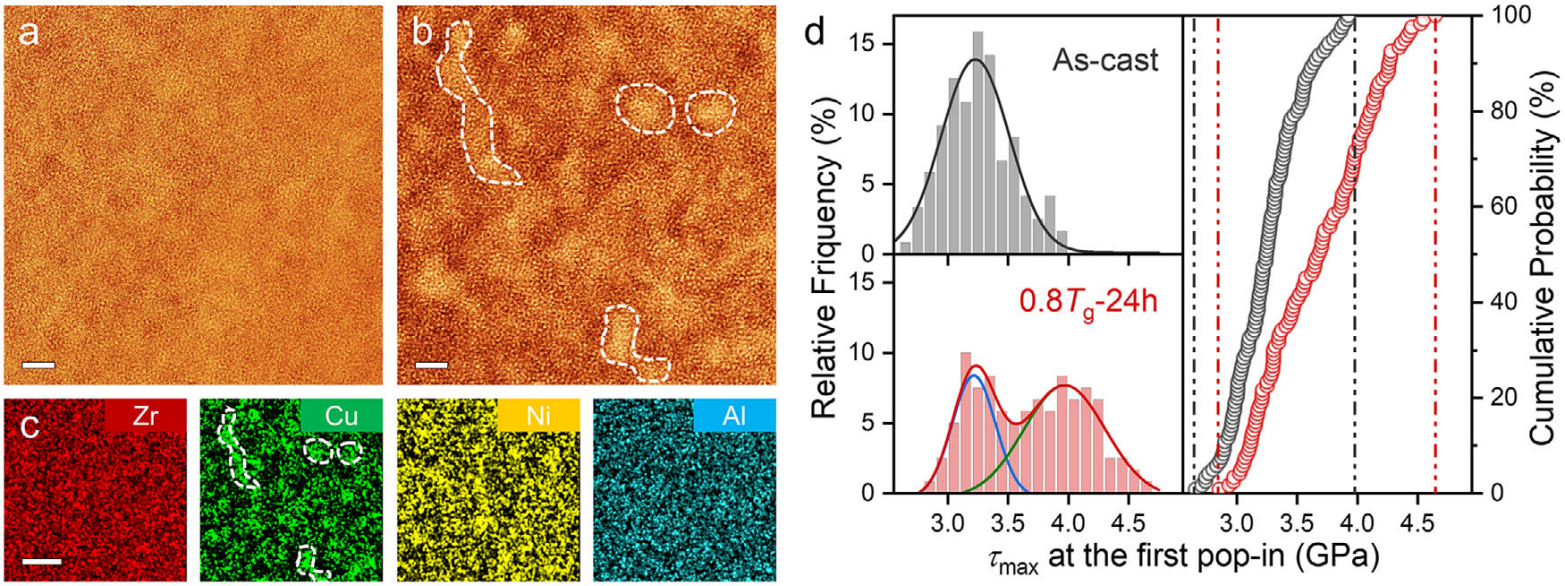
Microstructural evolution of Ni15 during annealing at 0.8 *T*_g_. (a,b) Cs‐corrected HAADF–STEM images of the as‐cast (a) and 24 h annealed alloys (b). HAADF contrast in (b) reveals enhanced chemical inhomogeneity. Scale bars: 2 nm. (c) EDS elemental maps showing clear spatial separation of Cu and Ni. Scale bar: 5 nm. (d) Statistical distribution of the maximum shear stress (*τ*_max_) probed by nanoindentation: relative frequency histograms (left) for the as‐cast (top) and 24 h annealed (bottom) alloys, and the corresponding cumulative probability plots (right). The annealed alloy exhibits a bimodal distribution (bottom left) fitted with a double‐Gaussian mixture, evidencing two distinct populations of shear‐active sites. Number of indents: *n* = 120 per condition.

A pivotal insight emerged when we discovered that annealing does not homogenize the glass; instead, it actively engineers a novel form of heterogeneity. High‐angle annular dark‐field scanning transmission electron microscopy (HAADF‐STEM) with spherical aberration correction uncovered pronounced nanoscale contrast variations after 24 h annealing at 0.8 *T*_g_ (Figure 2a,b), signaling amplified chemical and density fluctuations. Strikingly, this intensification opposes the usual contrast fade reported for many other Zr‐based glasses upon annealing [34]. Amplitude‐modulation atomic force microscopy (AFM) [34, 35, 36] data further corroborate this observation: phase‐shift mapping, which probes subsurface mechanical variations, demonstrated the structural correlation length growing from 7.29 ± 0.15 nm in the as‐cast state to 11.30 ± 0.17 nm after annealing (Figure S7), indicating expanded spatial heterogeneity. In contrast, reference Ni0 BMG exhibited the expected decrease in correlation length under identical treatment [34, 37, 38] (Figure S8), highlighting that Ni15 follows an anomalous, heterogeneity‐amplifying pathway.

Atom probe tomography (APT) frequency distribution analysis [39, 40] revealed that annealing induced significant deviations from random distribution for Zr, Cu, and Ni, whilst Al remained uniformly distributed (Figure S9 and Table S2 and Note S2). This statistical evidence for nanoscale clustering was visually corroborated by STEM‐EDS mapping (Figure 2c), which illustrated heterogeneous distribution of Cu and Ni, forming distinct Cu‐ and Ni‐rich domains amidst uniform Zr and Al backgrounds. Thus, the heterogeneity in Ni15 does not stem from reduction of quenched‐in disorder, but an active, chemistry‐guided amplification of compositional fluctuations during annealing.

The mechanical signature of this annealing‐engineered microstructure was captured by statistical nanoindentation, which probes the distribution of the maximum shear stress (*τ*_max_) at the first pop‐in event, signaling the onset of local plasticity [41, 42, 43]. While annealing typically narrows the *τ*_max_ distribution in conventional BMGs by annihilating weak LLPRs (Figure S10), the response of Ni15 is profoundly different. After the 24 h anneal, its *τ*_max_ distribution broadens significantly and develops a distinct bimodality, with a substantial portion of events shifting to higher stresses (Figure 2d). This bimodal pattern provides a statistical signature of distinct local mechanical environments. We interpret this as the direct mechanical response of a structural duality: it reflects the depletion of low‐stress sites (the suppressed low‐*τ*_max_ tail) and the emergence of a new population of higher‐stress sites (the new high‐*τ*_max_ peak). This mechanical heterogeneity is consistent with the chemical fluctuations observed via EDS and APT.

Thus, the anomalous toughening of Ni15 is rooted in an annealing‐engineered heterogeneous landscape. Our multi‐scale characterization identifies a structural duality: the population of conventional plasticity carriers (LLPRs) is reduced, whereas a new population of sites with higher shear resistance is generated. While a definitive, direct probe of local packing density on the scale of several nanometers remains challenging, the observed chemical separation (Figure 2; Figure S9f) and the correlated fluctuation in atomic number density (Figure S11) strongly implies concomitant variation in local packing, based on the established principle that chemical variation with atomic size mismatch governs local atomic packing and free volume [44, 45, 46]. A pivotal question, however, remains: can these newly formed, higher‐stress regions (LDPRs) actively participate in plastic flow and thereby compensate for the lost LLPRs? The experimental evidence points toward this possibility but cannot confirm it. We therefore proceed to test the hypothesis through atomistic simulations.

### 2.3 A Compensatory Plasticity Mechanism

To unravel the atomistic origin of compensatory plasticity and to validate the hypothesis emerging from our experiments, we employed molecular dynamics (MD) and Monte Carlo (MC) simulations. We generated a realistic Zr_64_Cu_21_Ni_15_ metallic glass model that captures the essential chemistry of Ni15 alloy (Figure S12). Although the simulated compressive stress–strain curves (Figure S13) differ quantitatively from experiments due to timescale limitations, they still captured the qualitative yield and flow behavior, providing a reliable basis for analyzing atomic‐scale deformation localization.

**FIGURE 3:**
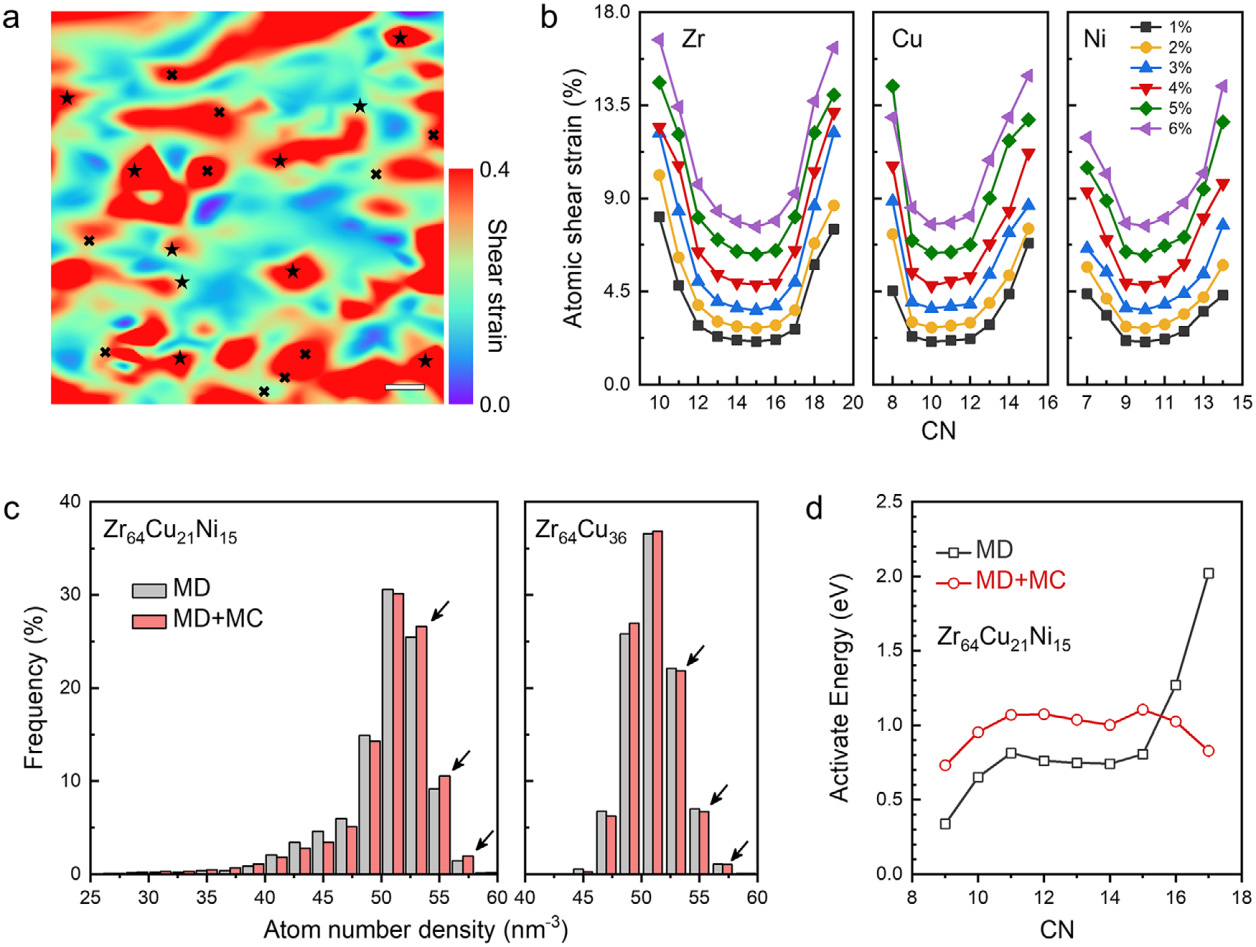
Structural and mechanical evolution of Zr_64_Cu_21_Ni_15_ from atomistic simulation. (a) Atomic shear strain mapping at 5% compressive strain. Crosses and pentagrams denote the ten atoms with the lowest and highest CN in the cross‐section, respectively, illustrating strain localization at both packing extremes. Scale bars: 0.5 nm. (b) U‐shaped dependence of the average local atomic shear strain on CN for all constituent elements (Zr, Cu, and Ni). (c) Atomic number density distribution from MD and post‐MC relaxation. MC relaxation reduces low‐density regions while populating high‐density regions. The right panel shows the corresponding distribution for Zr─Cu alloy as a reference. (d) CN‐dependent activation energy for deformation before and after MC annealing. Annealing significantly reduces the activation energy, particularly in the high‐CN regime.

A direct visualization of the atomic strain field at 5% total strain (Figure 3a) reveals an intuitive yet critical physical picture. The map shows a direct spatial correspondence between regions of high local shear strain (depicted in red) and atoms possessing either the lowest or highest coordination numbers (marked by crosses and pentagrams, respectively), visually confirming that structural instability driving plasticity originates from both ends of the atomic packing spectrum.

To systematically quantify this behavior, we defined the average atomic shear strain at the macroscopic yield point (4% strain) as a threshold, classifying atoms exceeding this value as high‐strain sites. After categorizing these atoms by type and coordination number (CN), we calculated the fraction of high‐strain atoms within each category relative to its total population. The resulting distribution of high‐strain atoms is distinctly U‐shape (Figure S14a), demonstrating a comparable propensity for plastic initiation in both low‐CN and high‐CN regions. To further verify this dual‐site activation mechanism, we plotted the average local shear strain of all non‐equilibrium atoms against their CN, which reproduces the same U‐shaped dependence (Figure 3b). Thus, atoms situated at either end of the packing spectrum carry the highest local strain and serve as the preferred sites for shear transformation. This provides atomic‐scale evidence that plastic flow is cooperatively mediated by loosely packed (low‐CN) and densely packed (high‐CN) regions.

The observed mechanistic picture is rationalized by the underlying atomic energy landscape. The atomic potential energy peaks at both extremes of the CN, as shown in Figure S14b. This U‐shaped energetic profile directly mirrors the plastic activation propensity in Figure 3a,b, demonstrating that deformation initiates preferentially at high‐energy atomic configurations, independent of their local packing density. In addition, Voronoi tessellation [47] analysis confirms a marked shift in atomic packing following annealing: low‐CN sites decrease while high‐CN LDPRs increase (Figure 3c). More critically, the activation energy for deforming these high‐CN atoms drops significantly after MC relaxation (Figure 3d), while their activation displacement increases (Figure S15). This combination of a lower energy barrier and a larger displacement renders the annealing‐induced LDPRs more deformable. As a result, these LDPRs in the annealed sample are transformed from potential obstacles into active, compensatory carriers of plasticity.

To unequivocally demonstrate the pivotal role of Ni, we compare the Ni15 alloy with a traditional, anneal‐embrittled Zr_64_Cu_36_ glass. In this Ni‐free benchmark, annealing primarily annihilates LLPRs with only a marginal increase in LDPRs (Figure 3c), resulting in a net reduction of shear sites and consequent embrittlement. In contrast, Ni15, the presence of Ni in our alloy introduces chemical frustration, which promotes the formation of readily activatable LDPRs, as evidenced by distinct changes in chemical short‐range order parameters (Figure S16). Thus, Ni alloying fundamentally alters the relaxation pathway, switching it from a destructive depletion of plasticity carriers to a constructive duality that unlocks the concurrent gains in strength and ductility.

**FIGURE 4:**
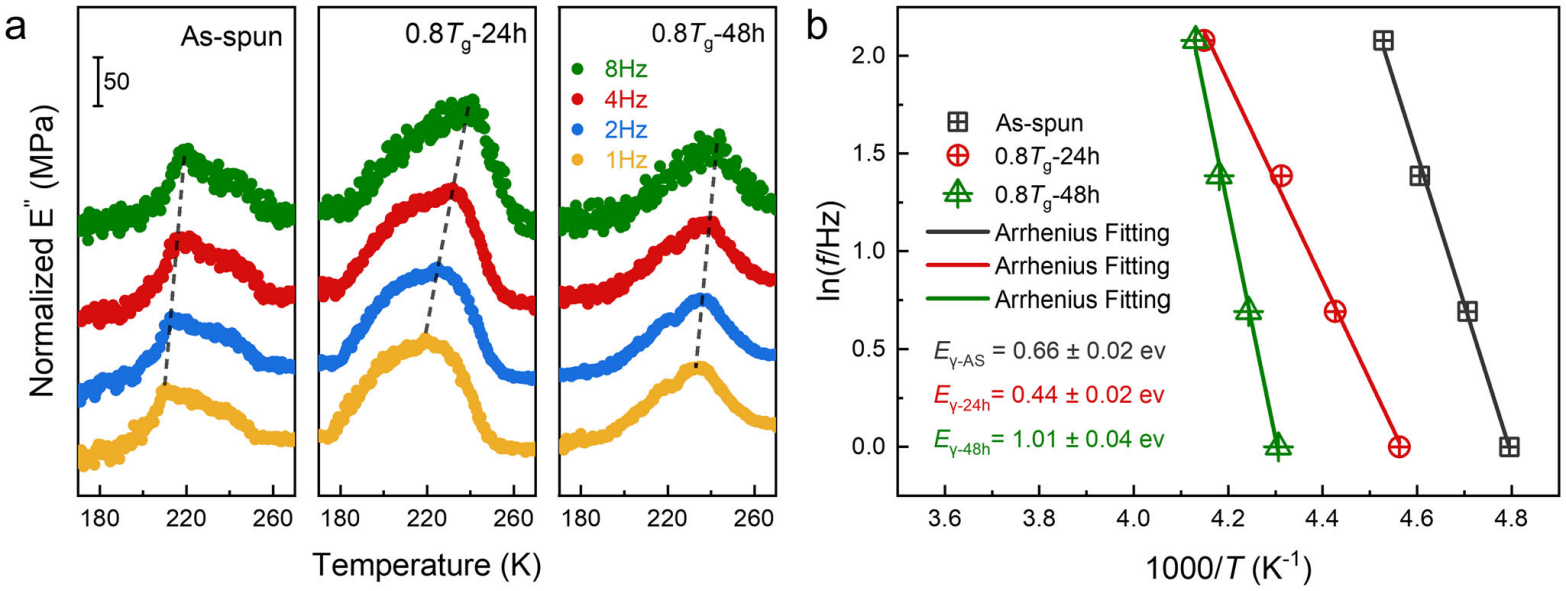
Reduced activation energy of *γ* relaxation in Ni15 after optimal annealing. (a) Isochronal relaxation spectra of the as‐spun, 24 h annealed (optimal duration), and 48 h annealed Ni15 ribbons were measured between 170 and 270 K at frequencies of 1, 2, 4, and 8 Hz. The 24 h annealed sample exhibits the most pronounced *γ* relaxation peak. (b) The corresponding activation energy of the *γ* relaxation drops sharply after 24 h of annealing in Ni15, indicating accelerated atomic mobility from optimized structural relaxation.

This atomic‐scale scenario in which a new low‐barrier plasticity carrier emerges predicts a global drop in the activation energy required to trigger plastic flow. Dynamic mechanical analysis (DMA) provides direct experimental validation: the activation energy (*E_γ_*) for the *γ* relaxation, a local mode that acts as a direct precursor to irreversible shear deformation [48, 49], drops to 0.44 ± 0.02 eV in the optimally annealed Ni15 alloy (Figure 4). This measured reduction corroborates the simulation prediction and confirms that the annealing‐induced structure indeed facilitates initiation of plastic events.

As elaborated above, the combined data yield a unified mechanism: Ni enables an annealing‐induced structural transformation in which newly formed, deformable LDPRs actively compensate for the lost LLPRs. This chemistry‐guided ordering breaks the long‐standing embrittlement paradigm by creating a heterogeneous energy landscape that lets plastic strain spread homogeneously, rather than collapsing into catastrophic shear bands.

## 3 Discussion

The entrenched view that structural relaxation inevitably embrittles MGs is overturned here. Sub‐*T*_g_ annealing of a Zr‐based bulk metallic glass simultaneously increases both strength and ductility (>150%), a behaviour irreconcilable with the classical free‐volume picture. The critical factor is a chemically engineered heterogeneity in which LLPRs and LDPRs coexist and deliver compensatory plasticity. As LLPRs vanish during relaxation, deformable LDPRs proliferate, preserving macroscopic flow.

The pursuit of a unified theory for plasticity in amorphous solids has yielded several influential concepts ranging from free‐volume [50], shear‐transformation‐zone models [51] to flow units [52, 53] and, more recently, to “soft spots” [25] that yield preferentially. Egami and co‐workers further proposed that both low‐ and high‐coordination environments in disordered systems are topologically unstable [24]. Our work builds directly upon this foundational insight. We provide the missing experimental and computational validation that plasticity indeed initiates preferentially at high‐energy atomic configurations located at both extremes of the packing spectrum. Beyond validating this unified picture, we crucially demonstrate how the principle can be actively engineered: through a targeted chemical design, we guide the spontaneous formation of deformable, high‐coordination sites (LDPRs) during annealing to act as compensatory plasticity carriers.

This energetic perspective clarifies why conventional ductilization strategies that primarily introduce LLPRs often sacrifice strength. A recent study showed that doping with small non‐metal atoms can also raise both strength and plasticity by seeding LDPRs [43]. Our thermal annealing approach, though methodologically distinct, converges on the same profound principle: deliberately create deformable LDPRs to act as potent and compensatory plasticity carriers. This agreement between disparate strategies underscores the robustness and universality of LDPR‐mediated toughening.

Our multi‐scale experimental evidence consistently traces this pathway. While DSC confirms free volume reduction, HAADF‐STEM and nanoindentation mapping reveal that chemical and density fluctuations are simultaneously amplified. AFM phase images corroborate the trend, in agreement with earlier observations on similar Zr‐based glasses by Liu et al. [35]. This annealing‐induced rise in heterogeneity stands in sharp contrast to the usual reduction reported for most MGs [34, 37, 38] and challenges the prevailing view of progressive degradation of heterogeneity during *β*‐relaxation [34]. The bimodal distribution of pop‐in shear stress furnishes the direct mechanical signature of the resulting structural duality.

This self‐organized compensation originates from a deliberate chemical design that exploits the positive enthalpy of mixing between Ni and Cu. This repulsive interaction creates a thermodynamic driving force for local atomic reorganization. This binary‐interaction‐dominated process is further modulated by ternary chemical preferences, consistent with the complex interplay highlighted in recent studies of multicomponent metallic glasses [54]. Cu possesses the highest positive mixing enthalpy [55, 56] with the other constituents and is the most prone to redistribution. During annealing at 0.8 *T*_g_, this driving force prompts nanoscale chemical partitioning, whilst the constrained kinetics confine the process to short‐range diffusion. The process is effective within a practical temperature window (∼0.8–0.9 *T*_g_), where the prominent *β*‐relaxation provides the necessary atomic mobility for redistribution [57, 58]. The result, evidenced by APT (Figure S9) and EDS, is the formation of Cu‐enriched regions where packing frustration templates the crucial, deformable LDPRs [45, 55, 59, 60]. Critically, this process exemplifies how a finely tuned thermodynamic driving force, coupled with restricted diffusion, can guide the glass into a targeted, non‐equilibrium state of beneficial heterogeneity.

This principle of guided structural evolution was systematically implemented through a design framework comprising three key aspects. First, in composition design, we employed a Zr‐based matrix (∼60 at.% Zr) (Figure S17) centred on a Ni─Cu pair with a good glass‐forming ability [61, 62, 63, 64, 65, 66]. The Ni─Cu pair provides the thermodynamic drive (positive mixing enthalpy) and templates the LDPR structure [53, 54, 55]. Second, in processing, rapid quenching produced a homogeneous, metastable glassy precursor. Third, in structural guidance, sub‐*T*_g_ annealing triggered elemental redistribution, whose outcome critically depended on precisely tuning the Ni/Cu ratio. Within the optimal window of 10–20 at.% Ni, corresponding to the apex of the calculated spinodal region (Figure S17c), an interface‐free, spinodal‐like heterogeneous structure was formed (Figure 2) [67]. In this optimally tuned state, the spontaneous generation of deformable LDPRs compensates for the loss of LLPRs, culminating in the observed annealing‐induced toughening (Figure S18). This may establish a general guideline for designing compensatory plasticity in other glass systems.

MD simulations confirm that this chemically guided structural evolution dictates the unprecedented plastic response. These simulations establish a fundamental principle: plasticity can initiate in both LLPRs and LDPRs because each sits at a high‐energy configuration. However, the decisive factor is how the glass responds to annealing. In traditional glasses, annealing primarily eliminates LLPRs without sufficiently generating new, deformable LDPRs, eventually leading to embrittlement. In our alloy, the specific chemistry design enables the self‐organized compensation described above: the population of LDPRs increases spontaneously, and their low activation barriers preserve high deformability; thus, the annealed microstructure offsets lost LLPRs with new, readily activated LDPRs.

While these atomistic simulations reveal key mechanistic precursors, such as the increased population of high‐coordination atoms and the short‐range tendency for Cu─Ni separation, they operate at scales too limited to directly form the nanoscale chemically heterogeneous domains observed after annealing (by TEM, APT, and STEM‐EDS). These larger domains result from annealing‐induced phase separation following a spinodal‐like pathway (Figure S17). The chemical driving force and deformable dense regions identified in simulations are the essential precursors that, if amplified by long‐range diffusion during actual annealing, would lead to the self‐organized nanostructure.

This atomic‐scale scenario makes a critical prediction: the global energy barrier for initiating plastic flow should decrease. Our dynamic mechanical analysis provides definitive experimental validation: the activation energy (*E*_γ_) for the *γ* relaxation drops to its lowest value in the optimally annealed alloy. The same trend is captured by MD simulations, which show a marked reduction in the shear‐activation barrier at high‐coordination sites. The quantitative agreement links the macroscopic energy landscape measured by DMA to the nanometre‐scale rearrangements revealed by simulation, validating the LDPR‐mediated compensatory mechanism. These findings provide a close connection between the global energy landscape and local atomic rearrangements.

## 4 Conclusion

In conclusion, we have established that annealing‐induced embrittlement is not an inevitable consequence of structural relaxation, but rather the outcome of a specific evolutionary path that can be deliberately redirected. By harnessing the positive Ni─Cu mixing enthalpy, sub‐*T*_g_ annealing of a Zr‐based BMG simultaneously enhances strength and plasticity. Our multi‐scale characterization reveals nanoscale chemical heterogeneity, while atomistic simulations suggest that the alloy chemistry promotes deformable LDPRs with reduced activation barriers. These LDPRs may contribute to the observed compensatory plasticity, though the precise relationship between atomic‐scale packing fluctuations and the nanoscale chemical domains requires further investigation. This work demonstrates that thermal processing can be transformed into a means of microstructural design, opening a route to toughen glassy materials through engineering of heterogeneous structures.

## 5 Experimental Methods

### 5.1 Materials Preparation

Bulk metallic glasses with nominal composition Zr_60_Cu_20_Ni_15_Al_5_ and Zr_60_Cu_30_Al_10_ (at.%) were prepared by arc‐melting mixtures of pure Zr, Cu, Ni, and Al metals (purity >99.99 wt.%) under a titanium‐gettered high‐purity argon atmosphere. To ensure chemical homogeneity, the ingots were remelted at least five times. Glassy rods were then fabricated by injection casting the molten master alloy into a water‐cooled copper mold under an argon atmosphere.

### 5.2 Thermodynamics and Properties Measurements

Thermal properties were characterized using a differential scanning calorimeter (NETZSCH DSC 404 F1). Samples of approximately 15–20 mg were encapsulated in aluminium pans under an inert atmosphere. DSC scans were performed at a constant heating rate of 10 K/min from room temperature to 873 K. The enthalpy change (∆*H*) associated with the relaxation exotherm in the glass transition region (400–650 K) was quantified by integrating the heat flow curve after baseline correction. Values of ∆*H* were normalized to those of the as‐cast sample (∆*H*_as‐cast_). To describe the isothermal structural relaxation behavior, the normalized enthalpy change ∆*H*/∆*H*_as‐cast_ was modeled using the Kohlrausch–Williams–Watts (KWW) stretched exponential function [33]: Δ*H*(*t_a_*)  =  Δ*H*_0_ + (Δ*H*_*as* − *cast*_ − Δ*H*_0_)exp [− (*t_a_*/τ)^β^], where *t*_a_ is annealing time, ∆*H*_0_ denotes the enthalpy of the fully relaxed state, *τ* is the characteristic relaxation time, and *β* represents the stretching exponent. A nonlinear least‐squares fitting procedure was applied to determine ∆*H*_0_, *τ*, and *β*. The quality of the fit was evaluated using the coefficient of determination (*R* ^2^).

Compression tests were performed on cylindrical samples (4 mm in length and 2 mm in diameter, yielding an aspect ratio of 2:1) using a CMT4305 universal testing machine at an engineering strain rate of 2 × 10^−4^ s^−1^. Strain was measured and calibrated using a miniature strain gauge attached to the sample during loading.

Relaxation behavior of the metallic glasses was characterized using a TA Instruments Q800 dynamic mechanical analyzer. DMA specimens (0.05 mm × 1.5 mm × 25 mm) were prepared by sectioning melt‐spun ribbons. The complex dynamic modulus, 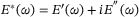, where *E*′ and *E*″ denote the storage and loss modulus, respectively, was measured in tensile mode. Tests were performed at a constant heating rate of 1 K/min and discrete frequencies of 1, 2, 4, and 8 Hz. The frequency dependence of the peak temperature for the γ‐relaxation process was described by the Arrhenius equation: $f=f_{0}\exp\left(-\frac{E_{\gamma }}{kT}\right)$, where *f*_0_ is a pre‐exponential factor and *E*_γ_ is the activation energy.

Nanoindentation measurements were performed to evaluate heterogeneity using an MTS DCM system equipped with a spherical diamond indenter (tip radius *R* = 1 µm). Tests were conducted at a constant strain rate of 0.05 s^−1^ to a maximum depth of 1000 nm. A minimum of 120 indents were performed on each sample to ensure statistically significant analysis of the first pop‐in events. The initial elastic loading segment follows the Hertzian contact model: $P=\frac{3}{4}E_{r}\sqrt{R}h^{\frac{3}{2}}$, where *P* is the load, *E*_r_ is the reduced modulus, *R* is the indenter radius, and *h* is the displacement. The reduced modulus is given by: $E_{r}={\left[\frac{1-v_{s}^{2}}{E_{s}}+\frac{1-v_{i}^{2}}{E_{i}}\right]}^{-1}$, where *E*_i_ = 1141 GPa and *v*_i_ = 0.07 are the Young's modulus and Poisson's ratio of the diamond indenter, and *E*_s_ and *v*_s_ are those of the sample. The corresponding maximum shear stress, occurring at a depth of approximately half the contact radius beneath the indenter, is given by: $\tau_{\max}=0.445{\left(\frac{16P_{pop-in}E_{r}^{2}}{9\pi^{3}R^{2}}\right)}^{\frac{1}{3}}$

### 5.3 Structure Characterization

The microstructural features and morphological details were examined using a Zeiss Supra‐55 field‐emission scanning electron microscope equipped with an energy‐dispersive X‐ray spectrometer. Transmission electron microscopy investigations were conducted on a JEOL JEM‐2200FS instrument. Atomic‐scale structural analysis was carried out using an aberration‐corrected FEI Titan Themis G2 microscope, which offers a spatial resolution of up to 60 pm. Specimens for TEM were prepared by mechanical grinding to a thickness of approximately 50 µm, followed by twin‐jet electropolishing with a solution of 25% nitric acid in methanol. The amorphous nature of the samples was verified by X‐ray diffraction (XRD; Rigaku Dmax‐RB) using Cu Kα radiation.

Atom probe tomography and three‐dimensional elemental mapping were conducted using a CAMECA LEAP 5000XR system. Analyses were performed in voltage‐pulsing mode at a specimen temperature of 50 K, with a pulse repetition rate of 200 kHz, a pulse fraction of 20%, and a detection rate of 0.5 ions per pulse. Data reconstruction, compositional analysis, and the generation of 2D distribution maps were carried out using Imago Visualization and Analysis Software (IVAS) version 3.8.0. APT needle specimens were prepared via focused ion beam (FIB) milling in a dual‐beam FEI Helios 600 microscope.

Amplitude‐modulation atomic force microscopy was conducted on the as‐prepared hyper‐quenched and relaxed samples without surface treatment to preserve their native morphology. Measurements were performed in tapping mode using an Oxford Instruments Asylum Cypher VRS system, equipped with an Olympus AC160 silicon probe (spring constant ∼26 N/m, resonant frequency ∼300 kHz). Height, phase, and amplitude images were acquired simultaneously under a set‐point amplitude ratio of ∼0.8 to minimize tip wear and surface disturbance. Spatial heterogeneity was quantified by fitting the phase image data to the correlation function*P*(*r*)  =  2σ^2^[1 − exp(− (*r*/ξ)^2α^)], where *ξ* denotes the lateral correlation length [34, 35].

### 5.4 Simulations

The Zr_64_Cu_21_Ni_15_ and Zr_64_Cu_36_ (at.%) metallic glass models were generated using MD simulations [68] with a trained deep learning potential [69] for the Zr─Cu─Ni system. Each system contained 13 500 atoms under periodic boundary conditions (PBCs). Simulations were conducted in the NPT ensemble with a time step of 2 fs, controlling temperature and pressure using a Nosé–Hoover thermostat and barostat [70]. The samples were first equilibrated at 300 K under zero external pressure for 1 ns, then heated to 2000 K and held for 1 ns to attain a fully liquid state. Subsequently, they were cooled to 300 K at a rate of 1 × 10^12^ K/s to form the glassy structure, followed by relaxation at 300 K for 1 ns. To prepare for activation–relaxation technique analyses [71], the samples were further cooled to 50 K at the same rate and relaxed for another 1 ns.

Annealing simulations were performed using a hybrid MC/MD approach. The samples were heated to 650 K (below the glass transition temperature, *T_g_*) and subjected to 100 000 MC steps for atomic exchange. A significant energy reduction was observed in the Zr_64_Cu_21_Ni_15_ system during this process, confirming the effectiveness of the MC relaxation. The systems were then cooled down to 300 K at 10^12^ K/s to obtain the final MC‐relaxed configurations.

For deformation simulations, a block model of Zr_64_Cu_21_Ni_15_ containing 27 000 atoms was prepared using the same quenching procedure and subsequently relaxed via the MC/MD method. Uniaxial pressing was applied along the *z*‐axis at a constant strain rate of 10^8^ s^−^^1^ at 300 K. During deformation, PBCs were maintained along the *z*‐direction, while free surfaces were introduced along the *x*‐ and *y*‐directions to accommodate shear offsets.

Thermodynamic calculations were performed with the CALPHAD [72] (Calculation of Phase Diagrams) approach implemented in the Pandat software (CompuTherm LLC) together with the PanHEA2020b_TH database.

## Author Contributions

Y.W. and Z.P.L. supervised the project and designed the experiments. X.J.L. designed the simulations. Y.J.Z., Y.H.C., J.Q.Y., Z.T., Z.C.L., F.S.L., Q.C., G.S.Z., and L.Q.S. synthesized and characterized the samples and performed the properties measurements. J.Y.W. and L.Q.L. carried out the simulations. Y.J.Z., J.Y.W., Y.W., X.J.L, and Z.P.L. wrote and revised the paper. All the authors, including Y.Y., H.W., S.H.J., and X.B.Z. analyzed the data and contributed to the discussion and interpretation of the results.

## Conflicts of Interest

The authors declare no conflicts of interest.

## Supporting information




**Supporting File**: advs74456‐sup‐0001‐SuppMat.docx.

## Data Availability

The data that support the findings of this study are available from the corresponding author upon reasonable request.
