# Supplementary material for: Annealing‐Induced Plasticity and Strengthening in Metallic Glasses
Source: Adv Sci (Weinh). 2026 Feb 23;13(24):e24294. doi: 10.1002/advs.202524294 (PMC13116290; doi:10.1002/advs.202524294)
Supplement: Supplementary file 1 — Supporting File: advs74456‐sup‐0001‐SuppMat.docx. [file ADVS-13-e24294-s001.docx]

Supporting Information

**Annealing-Induced Plasticity and Strengthening in Metallic Glasses**

Yingjie Zhang^1^, Jinyue Wang^1^, Leqing Liu^2^, Yuan Wu^1,3,4,^*, Yihuan Cao^1^, Jiqi Yu^5^, Zhen Tian^1^, Zhichao Lu^6^, Fengshou Li^1^, Qiang Chen^1^, Guosheng Zhang^1^, Laiquan Shen^5^, Yong Yu^1^, Hui Wang^1^, Suihe Jiang^1^, Xiaobin Zhang^1^, Xiongjun Liu^1,4,^* & Zhaoping Lu^1,^*

^1^ State Key Laboratory for Advanced Metals and Materials, University of Science and Technology Beijing, Beijing 100083, China

^2^ School of Intelligent Manufacturing and Mechanical Engineering, Hunan Institute of Technology, Hengyang 421002, China

^3^ School of Mathematics and Physics, University of Science and Technology Beijing, Beijing 100083, China

^4^ Institute for Materials Intelligent Technology, Liaoning Academy of Materials, Shenyang 110004, China

^5^ Institute of Physics, Chinese Academy of Sciences, Beijing 100190, China

^6^ Neutron Science Center, Songshan Lake Materials Laboratory, Dongguan 523800, China

(Yingjie Zhang and Jinyue Wang contributed equally to this work.)

* Corresponding author:

wuyuan@ustb.edu.cn (Y. Wu); xjliu@ustb.edu.cn (X.J. Liu); luzp@ustb.edu.cn (Z.P. Lu)

**Table S1. Summary of characteristic properties.** Mean values from three independent measurements of elastic constants determined by ultrasonic testing, including density (*ρ*), Young’s modulus (*E*), shear modulus (*G*), bulk modulus (*B*), and Poisson’s ratio (*ν*), for the investigated alloys.

|  | *ρ*(g/cm^3^) | *E*(GPa) | *G*(GPa) | *B*(GPa) | *v* | *G*/*B* |
| --- | --- | --- | --- | --- | --- | --- |
| As-cast | 6.71 | 75.9 | 27.8 | 94.5 | 0.37 | 0.294 |
| 0.8 *T*_g_-1h | 6.72 | 77.6 | 28.7 | 86.8 | 0.35 | 0.331 |
| 0.8 *T*_g_-12h | 6.82 | 80.6 | 29.4 | 105.0 | 0.37 | 0.280 |
| 0.8 *T*_g_ -24h | 6.83 | 81.0 | 29.5 | 107.6 | 0.37 | 0.274 |
| 0.8 *T*_g_-48h | 6.85 | 80.3 | 29.6 | 94.9 | 0.36 | 0.312 |


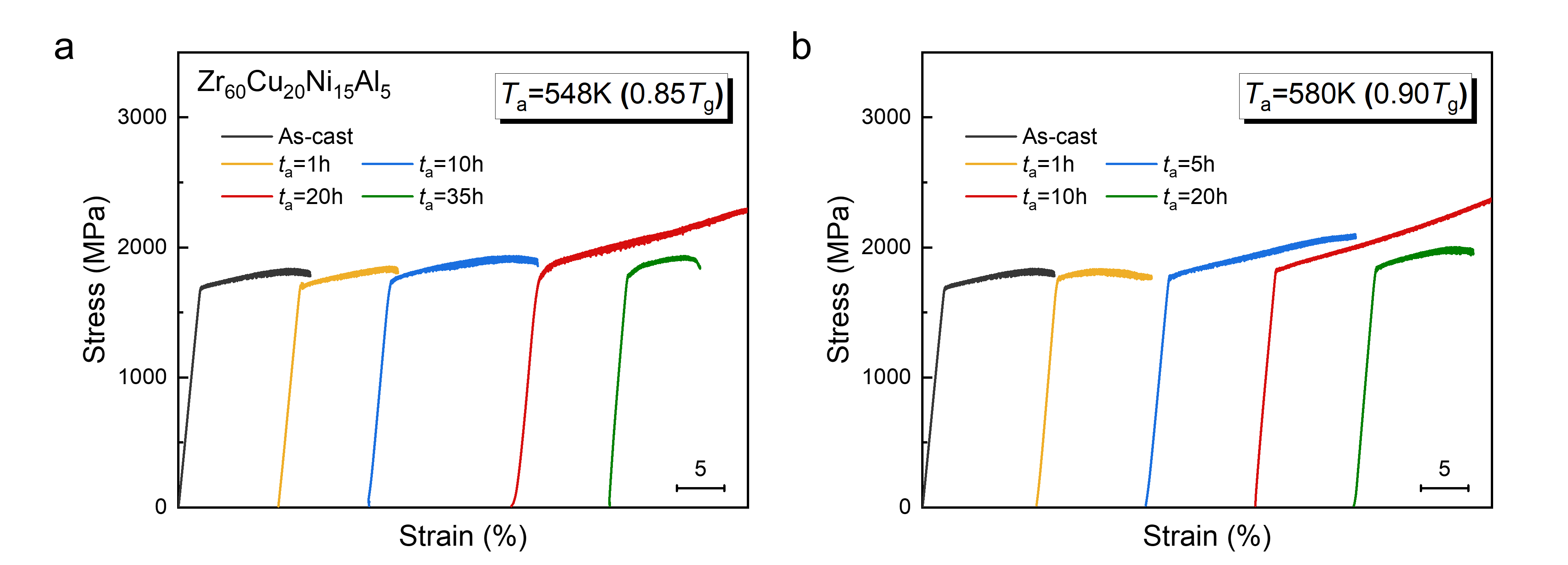


**Figure S1 Unified non-monotonic annealing response in Ni15 BMGs. (a)** Compressive engineering stress-strain curves of Ni15 BMGs annealed at 0.85 *T*_g_ for the indicated durations. **(b)** Corresponding curves at 0.90 *T*_g_. All temperatures induce identical non-monotonicity, replicating the 0.8 *T*_g_ response (Fig. 1) with accelerated kinetics at higher *T*_a_.


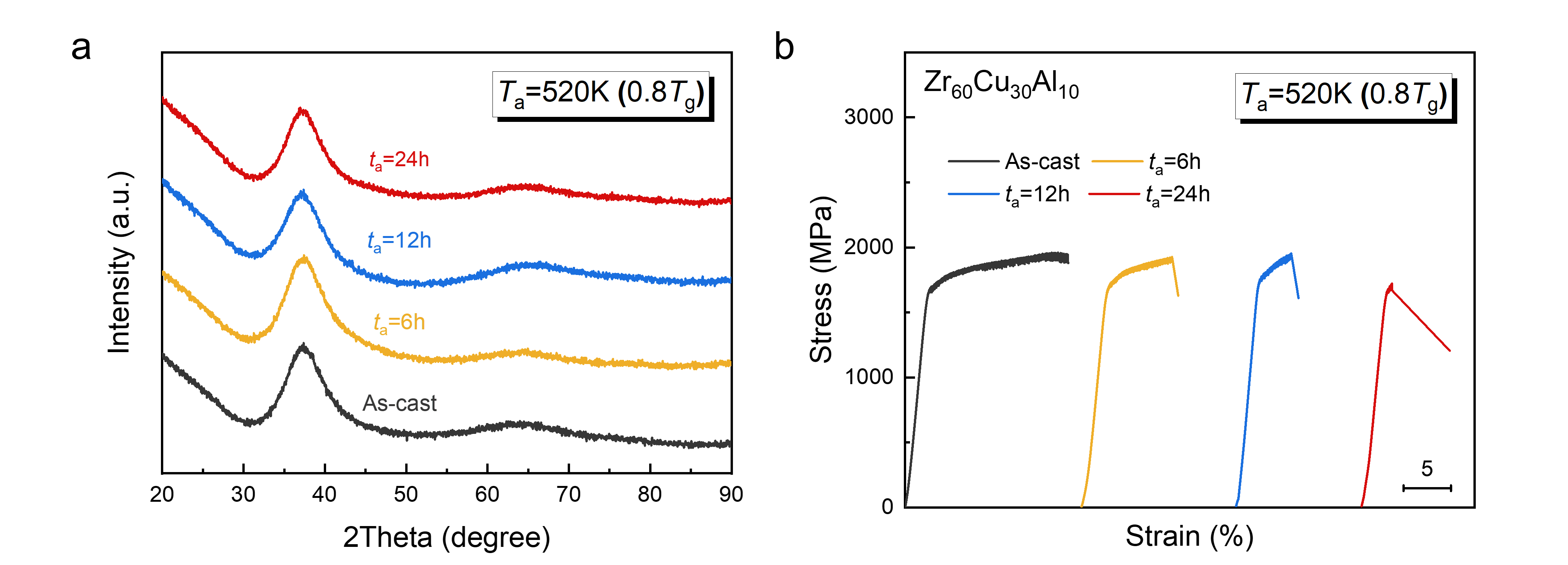


**Figure S2 Structural evolution and annealing-induced embrittlement in Al10 BMGs. (a)** XRD spectra of Al10 BMGs annealed at 0.8 *T*_g_ for the indicated durations. The broad diffraction halo at 2*θ* ≈ 30–45° confirms the amorphous nature. **(b)** Compressive engineering stress-strain curves of Al10 BMGs annealed under identical conditions, exhibiting progressive loss of plasticity and abrupt fracture, indicative of characteristic annealing embrittlement.

**
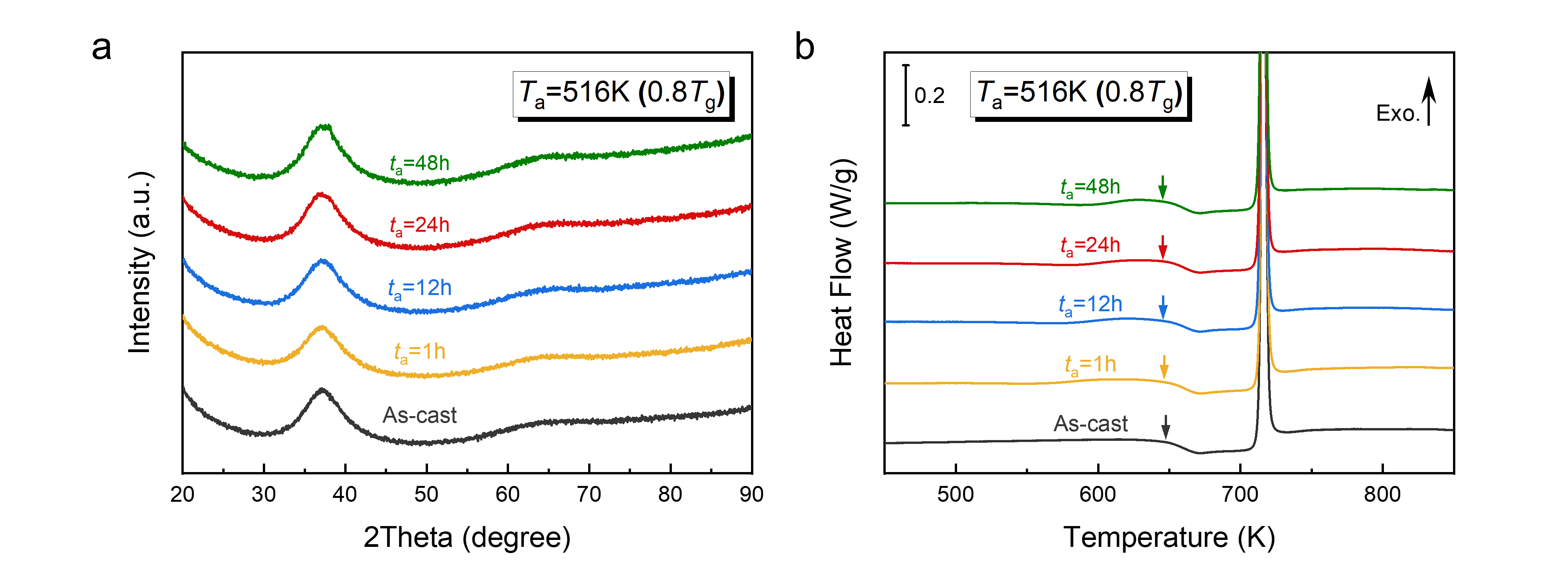
**

**Figure S3 (a)** XRD spectra of Ni15 BMGs annealed at 0.8 *T*_g_ for the indicated durations. The broad diffraction halo at 2*θ* ≈ 30–45°confirms the amorphous nature. **(b)** DSC traces recorded at a heating rate of 10 K/min reveal that both the glass transition and crystallization events remain largely unchanged with annealing time. Arrows indicate the glass transition temperature *T*_g_.

**
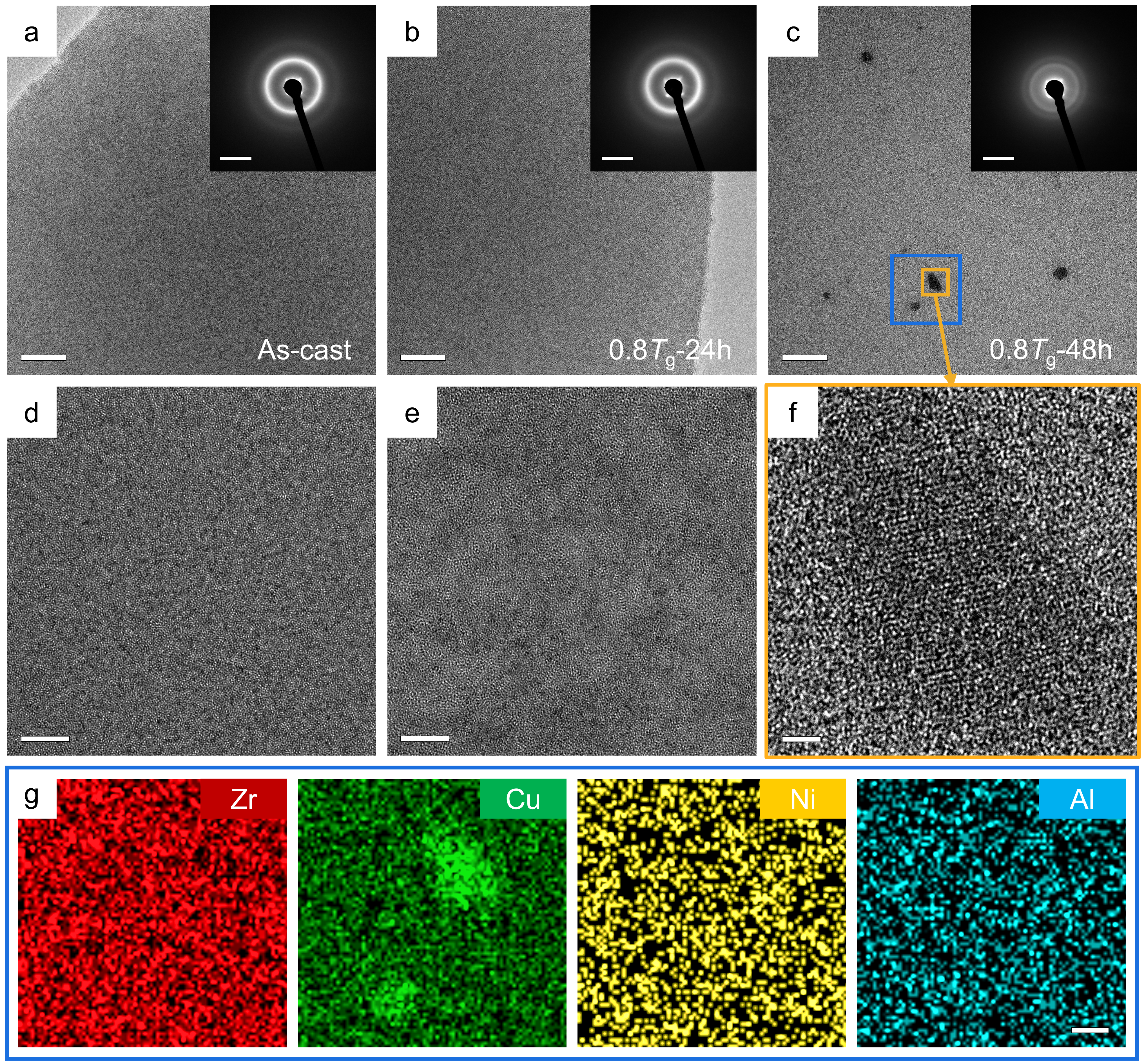
**

**Figure S4 Time-dependent microstructural evolution of Ni15 BMG annealed at 0.8 *T*_g_. (a–c)** Bright-field TEM images and the corresponding SAED patterns (insets) of (a) the as-cast, (b) 24 h-annealed, and (c) 48 h-annealed states. **Scale bars:** 50 nm (TEM), 5 nm⁻¹ (SAED). **(d–f)** HR-TEM images of (d) the as-cast matrix, (e) 24 h-annealed matrix, and (f) Nanocrystalline phase within the 48 h-annealed matrix (yellow rectangle in c). **Scale bars:** 5 nm (d, e), 2 nm (f). **(g)** EDS elemental maps of Zr, Cu, Ni and Al from the blue rectangular region in c. **Scale bar:** 10 nm.

**
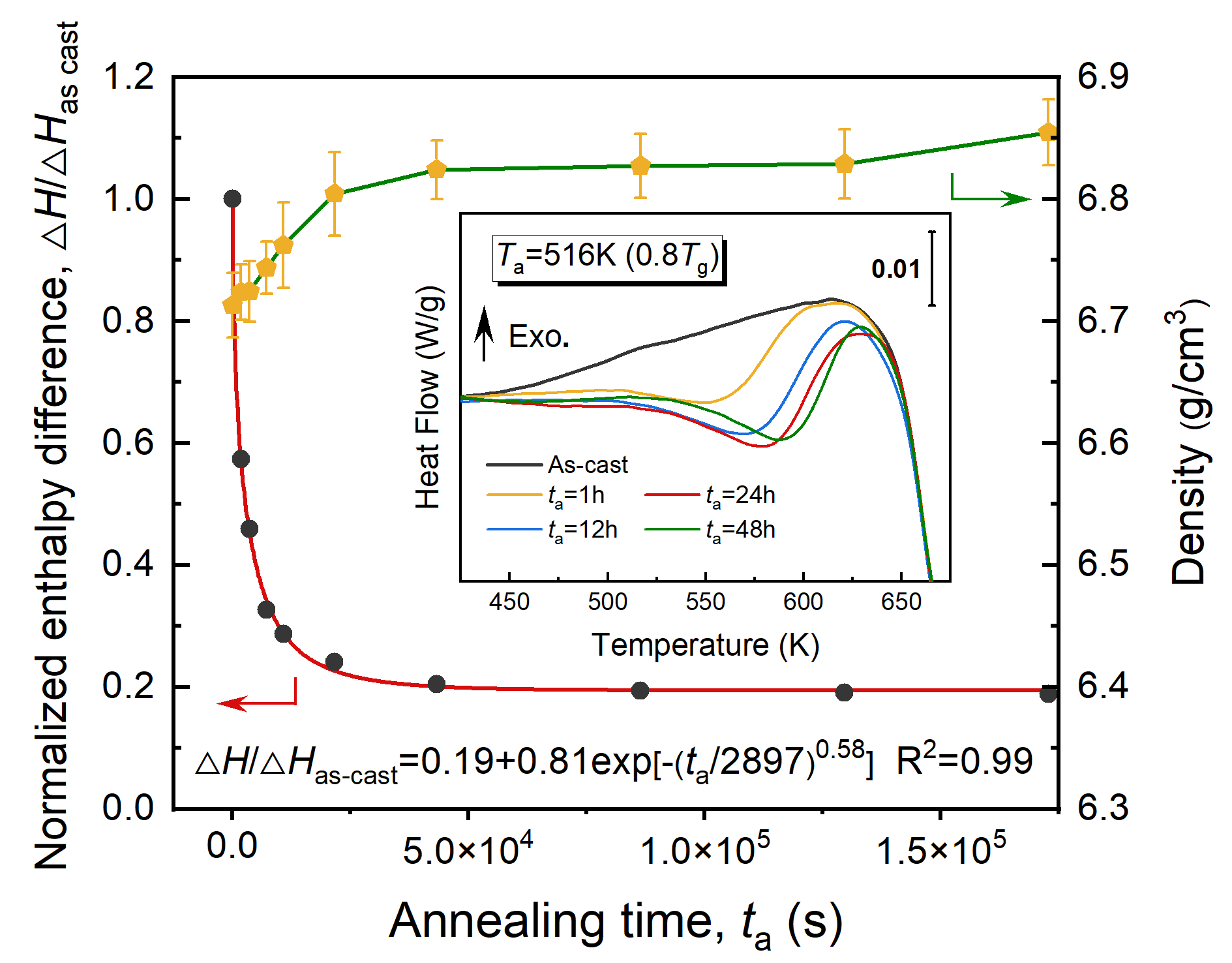
**

**Figure S5** Variation of the enthalpy difference (Δ*H*, derived from DSC) and density (*ρ*) with annealing time (*t*_a_) during isothermal annealing (*T*_a_ = 0.8 *T*_g_) of Ni15 BMG. The concurrent reduction in Δ*H* and increase in *ρ* correlate with structural relaxation. Inset: Enlarged view of the glass transition region on DSC curves (heating rate: 10 K/min).

**Note S1**

Typically, low-temperature annealing of BMGs annihilates free volume, also known as LLPRs, which tends to embrittle the materials^[1, 2]^. Although the DSC scans of Ni15 BMGs before and after annealing appear similar at first glance, the inset in Figure S5 unveils subtle yet consistent alterations at the glass transition point. A sharp exothermic peak preceding the glass transition is seen for the as-cast glass, whose height reduces systematically with *t*_a_, which is attributed to the annihilation of excess free volume^[2]^, ∆*V*_f_. Consequently, it is feasible to gauge the alterations in free volume during annealing by tracking ∆*H*, as proposed in literature^[2, 3]^. The enthalpy change, ∆*H*, associated with the exothermic peak was determined by integrating the heat flow, *q*, (DSC thermogram) near the glass transition range (400–650 K). The ∆*H* values for each sample were normalized against the ∆*H*_as-cast_ of the as-cast state and are represented as a function of the annealing time, *t*_a_, in Figure 3c. It is observed that the ratio ∆*H*/∆*H*_as-cast_ declines by 80% after a 24-hour annealing period.

The relaxation kinetics in MGs are often found to obey the stretched exponential or Kohlraush–Williams–Watts (KWW) relaxation function^[4]^ of the form,

$\Delta H\left(t_{a}\right)=\Delta H_{0}+\left(\Delta H_{as-cast}-\Delta H_{0}\right)exp\left[-\left(t_{a}/\tau\right)^{\beta}\right]$ (1)

where $\Delta H_{0}$ represents the enthalpy difference for a fully annealed sample, *τ* denotes the characteristic relaxation time, and *β* is the parameter indicative of non-exponential relaxation behavior. By fitting Equation (1) to the experimental data, with ∆*H*_0_, *τ* and *β* as adjustable variables, we obtained the values ∆*H*_0_ = 190 J/g, *τ* = 2897 s, and *β* = 0.58. The goodness-of-fit metric, denoted as *R*^2^, has a value of 0.99, which strongly suggests that Equation (1) accurately models the isothermal relaxation behavior observed in the BMG samples. The determined value of ∆*H*_0_ closely matches the enthalpy change measured after a 24-hour annealing period, suggesting that the relaxation process is essentially completed at this time. Thus, the free volume indeed decreased after annealing, which is the reason why the overall structural heterogeneity has shifted towards a higher level of critical shear stress. Additionally, it was found that density (Figure S5) and Vickers hardness (Figure S6) increased with the extension of annealing time, and after a certain period, they tended to stabilize, indicating that overall densification finished after the prolonged annealing.

**
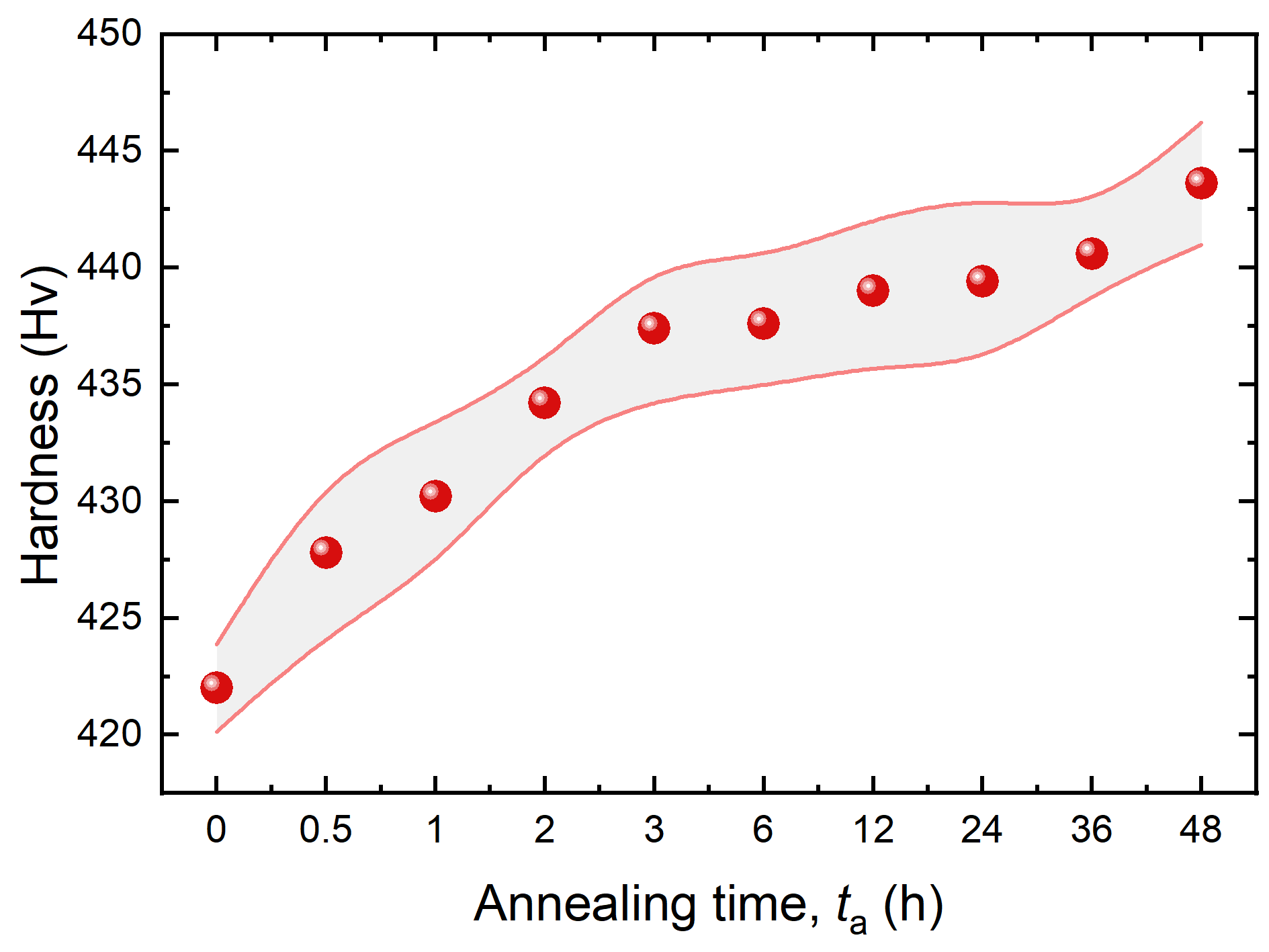
**

**Figure S6** Hardness of the Ni15 BMGs annealed at 0.8 *T*_g_ for different time intervals. The shaded region indicates the standard deviation of the mean.

**
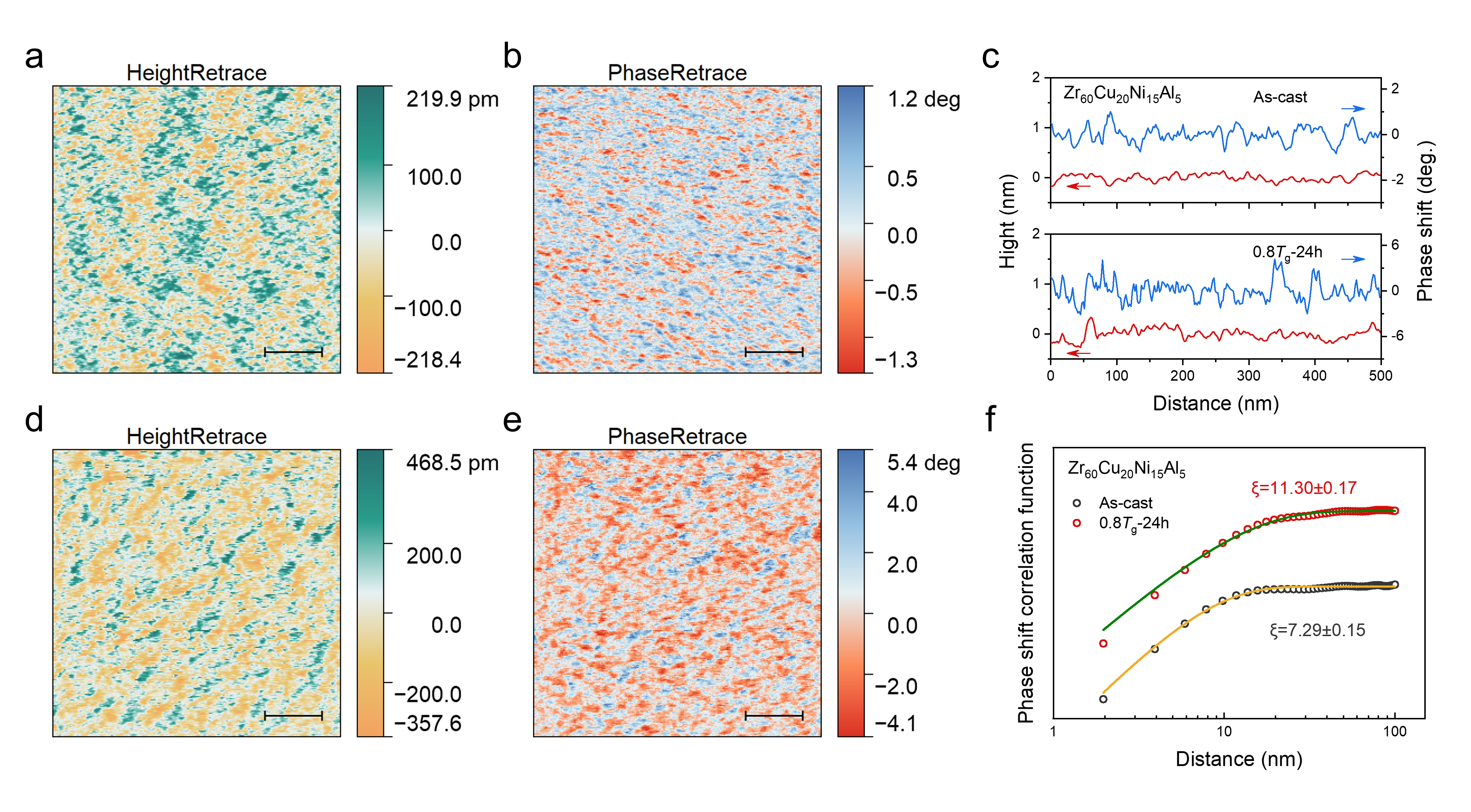
**

**Figure S7 AM-AFM analysis of nanoscale heterogeneity evolution in Ni15 BMGs. (a, b)** Height (a) and phase shift (b) images of the as-cast alloy. **(d, e)** Corresponding height (d) and phase shift (e) images of the 24h annealed alloy. Phase shift images reveal intensified nanoscale heterogeneity after annealing, reflecting enhanced compositional/mechanical fluctuations. **(c)** Line profiles confirm phase shift variations (blue) are decoupled from surface topography (red). **(f)** Quantified correlation lengths (*ξ*) of phase shift fields. Annealing increases *ξ* from 7.29 ± 0.15 nm (as-cast) to 11.30 ± 0.17 nm (24h), demonstrating amplified heterogeneity at nanoscale. **Scale bars:** 100 nm (a, b, d, e).

**
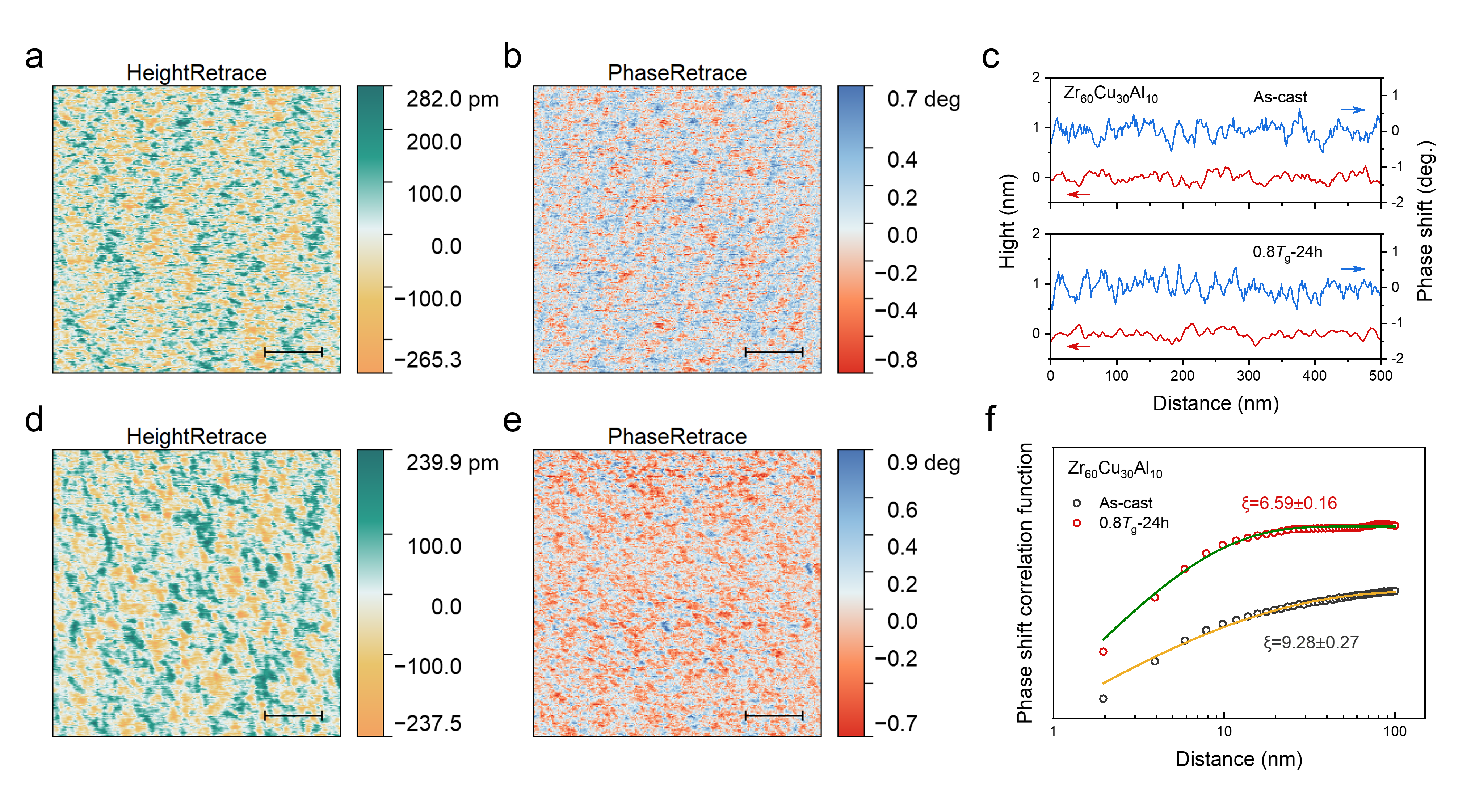
**

**Figure S8 AM-AFM analysis of nanoscale heterogeneity evolution in Al10 BMGs. (a, b)** Height (a) and phase shift (b) images of the as-cast alloy. **(d, e)** Corresponding height (d) and phase shift (e) images of the 24h annealed alloy. **(c)** Line profiles confirm phase shift variations (blue) are decoupled from surface topography (red). **(f)** Quantified correlation lengths (*ξ*) of phase shift fields: Annealing reduces *ξ* from 9.28 ± 0.27 nm (the as-cast) to 6.59 ± 0.16 nm (the 24 h-annealed), demonstrating homogenization at nanoscale. **Scale bars:** 100 nm (a, b, d, e).

**
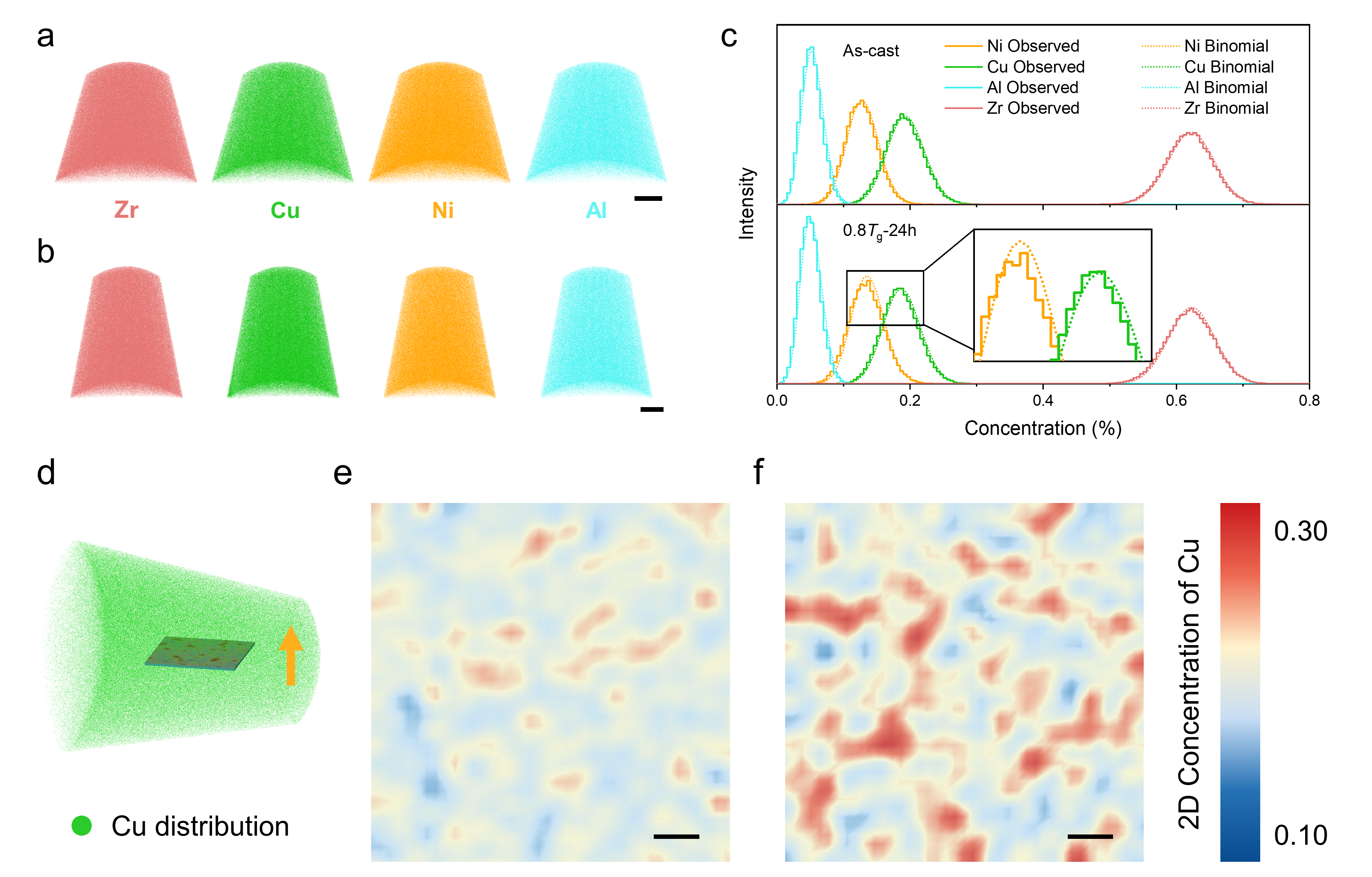
**

**Figure S9 APT quantification of elemental redistribution in Ni15 BMGs. (a, b)** Full-volume tomographic reconstructions showing macroscopically homogeneous elemental distributions in both the as-cast (a) and 24h annealed (b) states. **(c)** Atomic concentration distributions for the as-cast (top) and 24h annealed (bottom) samples. Solid lines represent experimental data while dashed lines denote the binomial distribution. Annealing drives significant deviation of Cu/Ni distributions from the binomial behavior, signifying formation of chemically ordered nanodomains. **(d, e, f)** 2D Cu concentration map over 40*40*1 nm^3^ volume of data for the as-cast (e) and 24h annealed samples (f), and their sampling schematic diagrams (d). **Scale bars:** 20 nm (a, b), 5 nm (e, f).

**Table S2. Binomial analysis of atomic concentration and the corresponding *p*-value and *μ* parameters in the as-cast and 24h annealed Ni15 alloys.** *p* < 0.01 is a commonly used standard value to reject the null hypothesis, indicating that there exists significant ordering within the material. *μ*, as a normalized auto-correlation parameter of *χ*^2^, ranges from 0 (random) to 1 (complete solute association).

|  | As-cast | | 0.8 *T*_g_-24h | |
| --- | --- | --- | --- | --- |
| Elements | *p*-value | *μ* | *p*-value | *μ* |
| Zr | 0.7995 | 0.0316 | <0.0001 | 0.1037 |
| Cu | 0.2287 | 0.0334 | <0.0001 | 0.1528 |
| Ni | 0.0845 | 0.0337 | <0.0001 | 0.1146 |
| Al | 0.1500 | 0.0264 | 0.0151 | 0.0248 |

**Note S2**

Frequency distribution analyses which can identify presence of nanoscale solute clusters and other segregation trends invisible in APT data sets, widely used to capture the earliest stage of phase decomposition in multicomponent alloys^[5, 6]^. We compared the frequency distribution of experimental APT data for Ni15 with that from a binomial distribution of the same mean concentration for each constituent, as shown in Figure S9c. Deviations of the experimental frequency distribution curves (solid curves in Figure S9c.) from binomial distribution functions (dashed curves in Figure S9c.) yield the *p* and *μ* values (Table S1). A *p* value of 0.01 is a commonly used standard to reject the null hypothesis in statistical hypothesis testing. Thus, if the *p* value is less than 0.01, the alloy is not homogeneous and elemental clustering within the alloy may prevail^[5]^. *μ* varies between 0 and 1, where 0 indicates a random distribution and 1 indicates a complete ordering of all constituent atoms^[5]^. For the as-cast alloy, *p* values significantly above 0.01 coupled with *μ* values close to zero, indicating a uniform elemental distribution. After 24 hours annealing, however, distribution of Zr, Cu and Ni deviated from the binomial curve, whilst the other elements still exhibited random distribution. This divergence is exemplified by a pair of 2D cross-sections in the form of Cu distribution maps provided in Figure S9e,f. Clearly, significant fluctuations in the Cu density are observed in the alloy after 24 hours annealing (Figure S9f), with pronounced red and dark blue regions.

**

**

**Figure S10 Statistical distribution of the maximum shear stress (*τ*_max_) of Ni0 measured by nanoindentation**. **Left**: relative frequency histograms for the as-cast (top) and 24h annealed (bottom) alloys; both are adequately fitted by single Gaussian fits. **Right**: the corresponding cumulative probability plots. Number of indents: n = 120 per condition.





**Figure S11 Number density fluctuation analysis via APT.** One-dimensional atom count profiles, normalized to the mean number density, were extracted from a cylindrical analysis volume (2 nm in diameter, 50 nm in length) within the APT reconstruction (sampling geometry in inset). The profile from the 24 h‑annealed specimen exhibits significantly larger fluctuations in atomic number density than the as‑cast state, revealing the development of pronounced nanoscale density heterogeneity during annealing.

**
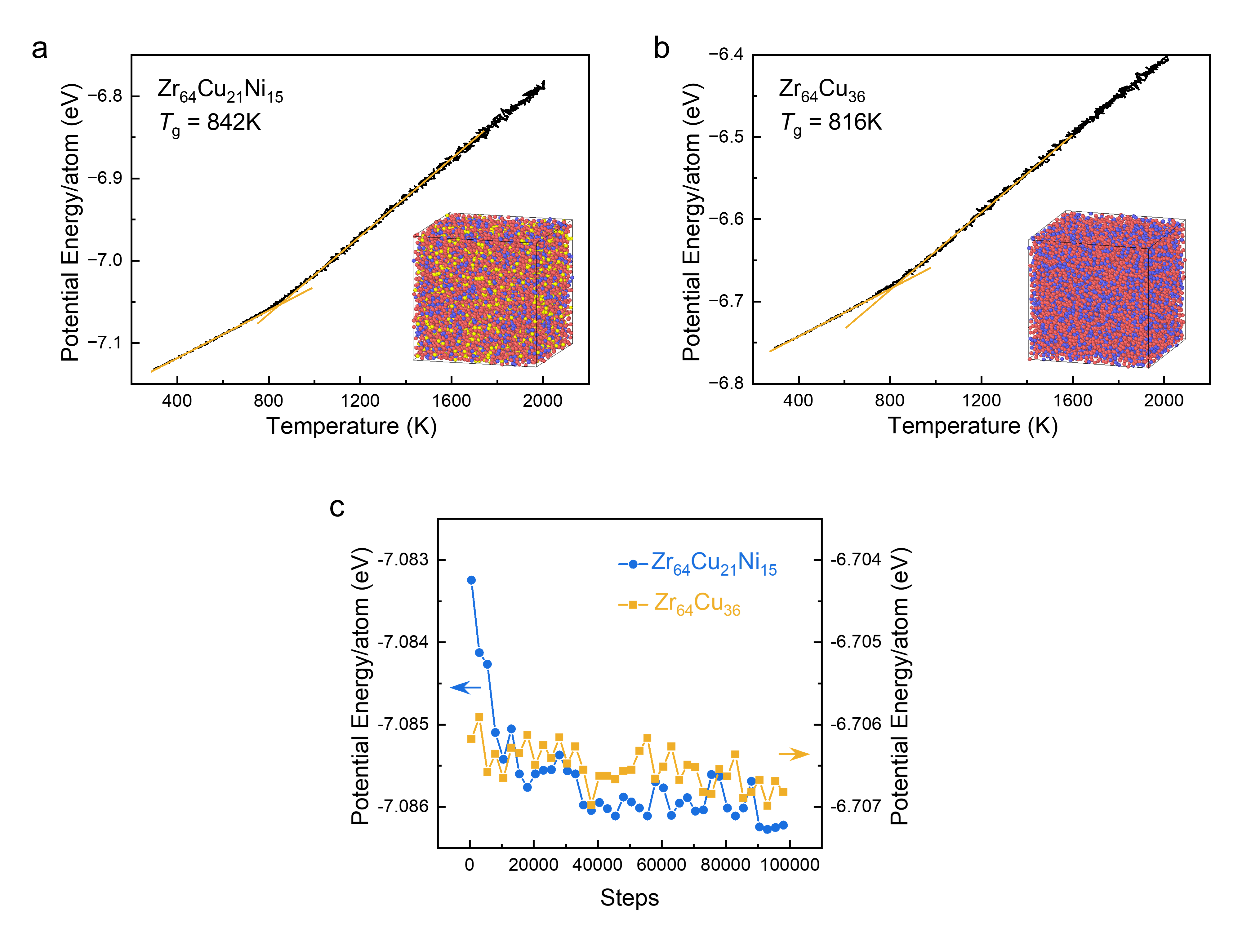
**

**Figure S12 Energy evolution during cooling and configurational exchange in Zr_64_Cu_36_ and Zr_64_Cu_21_Ni_15_ metallic glasses. (a, b)** Temperature dependence of the system energy for Zr_64_Cu_21_Ni_15_ (a) and Zr_64_Cu_36_ (b) during rapid cooling. The continuous evolution of energy with temperature, along with a distinct change in its temperature derivative near the *T*_g_, confirms formation of the amorphous phase. **(c)** Configurational evolution during Monte Carlo (MC) swaps between the two as-formed metallic glasses. A predominant exchange occurred between Cu and Ni in the Zr_64_Cu_21_Ni_15_ glass, whereas only 9 swapping steps were observed for Zr-Cu pairs. The energy of the Zr-Cu-Ni glass decreases significantly after MC swapping, whilst that of the Ni-free alloy remains essentially unchanged.

**

**

**Figure S13 Simulated compressive mechanical response of Zr_64_Cu_21_Ni_15_ from MD and MC simulations.** The simulated engineering stress-strain curves, while exhibiting quantitative deviations from experimental data, capture the qualitative trends critical to this study, thus validating the use of simulation models for the intended mechanistic analysis.

**
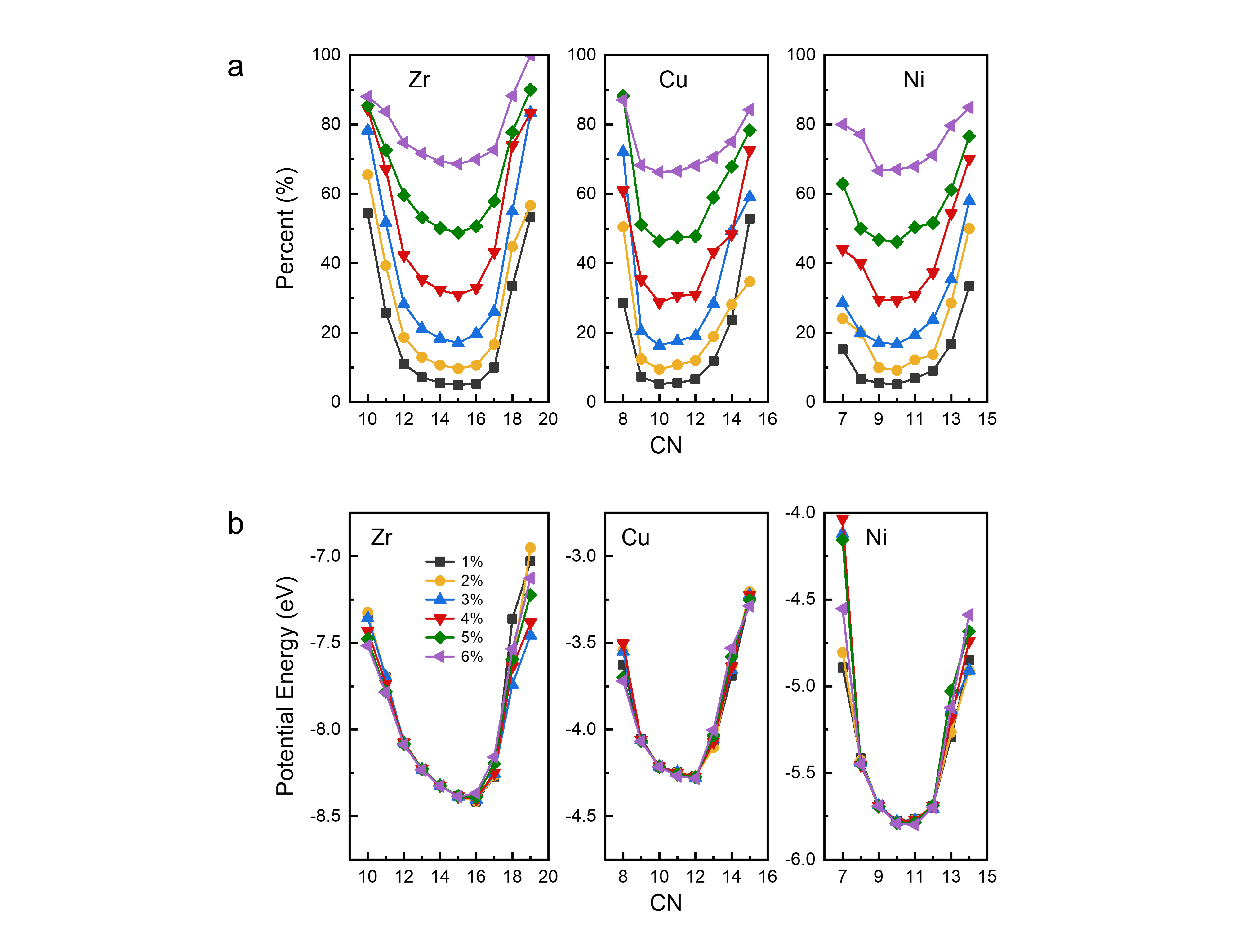
**

**Figure S14 All three constituent elements (Zr, Cu, Ni) under compression exhibit a characteristic U-shaped dependence on CN: (a)** percentage of high-strain atoms, **(b)** atomic potential energy.

**

**

**Figure S15 Activation displacement in Zr_64_Cu_21_Ni_15_ obtained from MD and MC simulations.** Larger values indicate a higher propensity for atomic activation under applied stress.

**
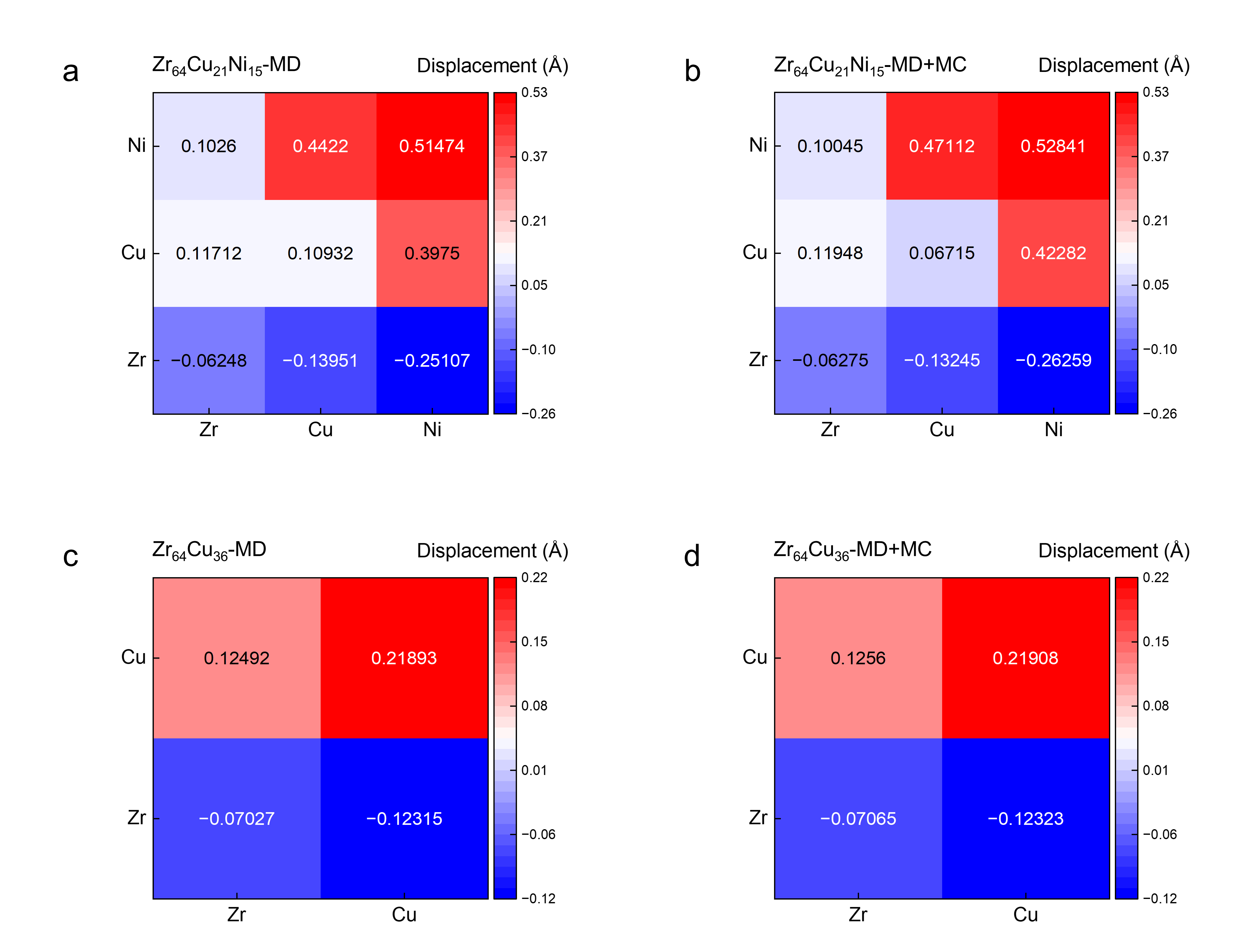
**

**Figure S16 Evolution of chemical short-range order (CSRO) parameters during MC swapping. (a, b)** Zr_64_Cu_21_Ni_15_ before (a) and after (b) MC swapping, showing a pronounced enhancement in Cu-Ni CSRO. **(c, d)** Zr_64_Cu_36_ before (c) and after (d) MC swapping, revealing negligible changes in its CSRO parameters. The contrast highlights the distinct configurational responses of the two alloys to the same MC protocol.

**
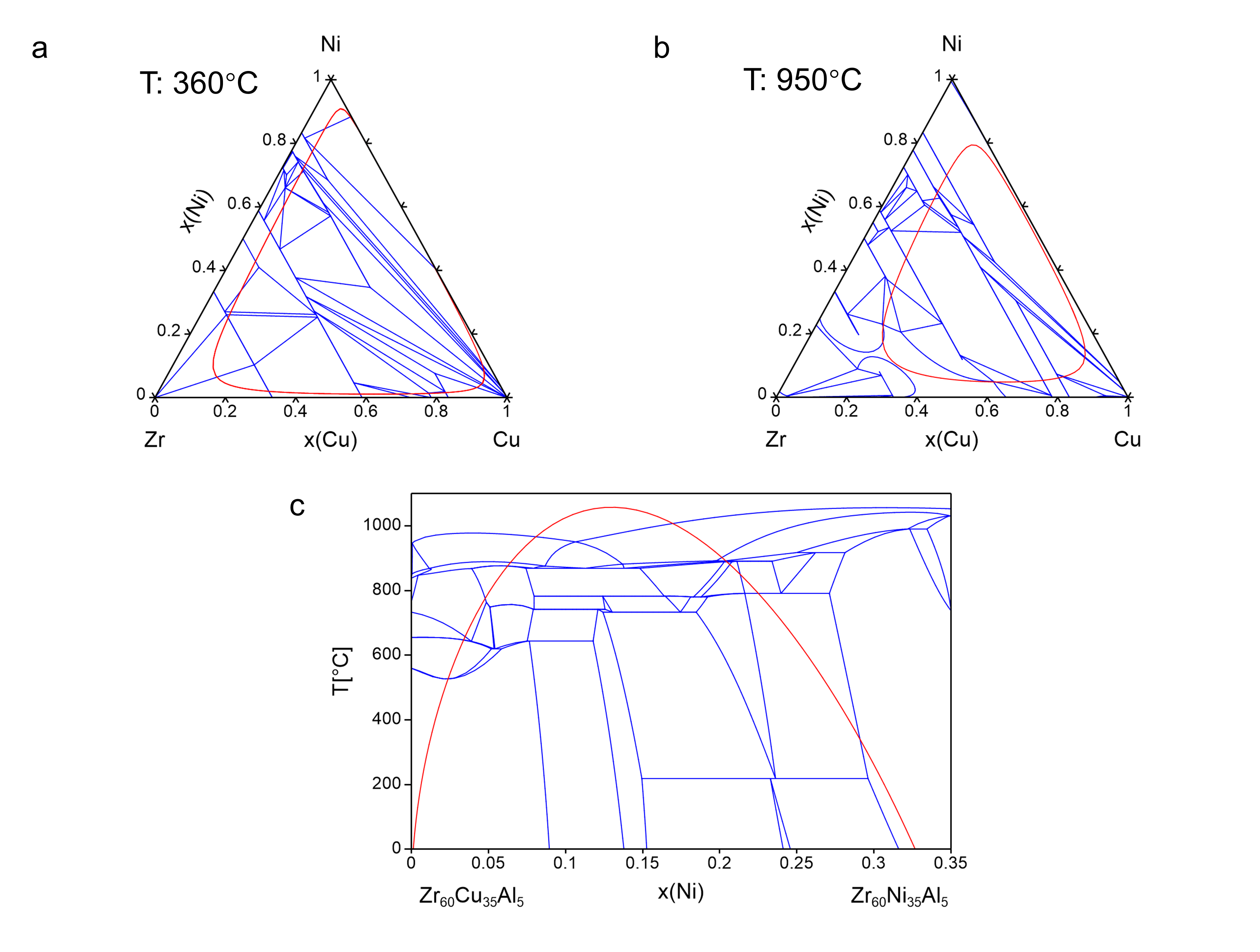
**

**Figure S17 CALPHAD-calculated phase diagrams for the Zr–Ni–Cu system.** **(a, b)** Isothermal sections of the Zr–Ni–Cu system calculated at (a) 360 °C (near *T*_g_) and (b) 950 °C (near the apex of the spinodal decomposition surface). **(c)** Simulated phase diagram for the composition cut between Zr_60_Cu_35_Al_5_ and Zr_60_Ni_35_Al_5_. The red line indicates the calculated spinodal curve, delineating the compositional range of metastable phase separation within this system. All phase diagrams were constructed by the CALPHAD method.

**
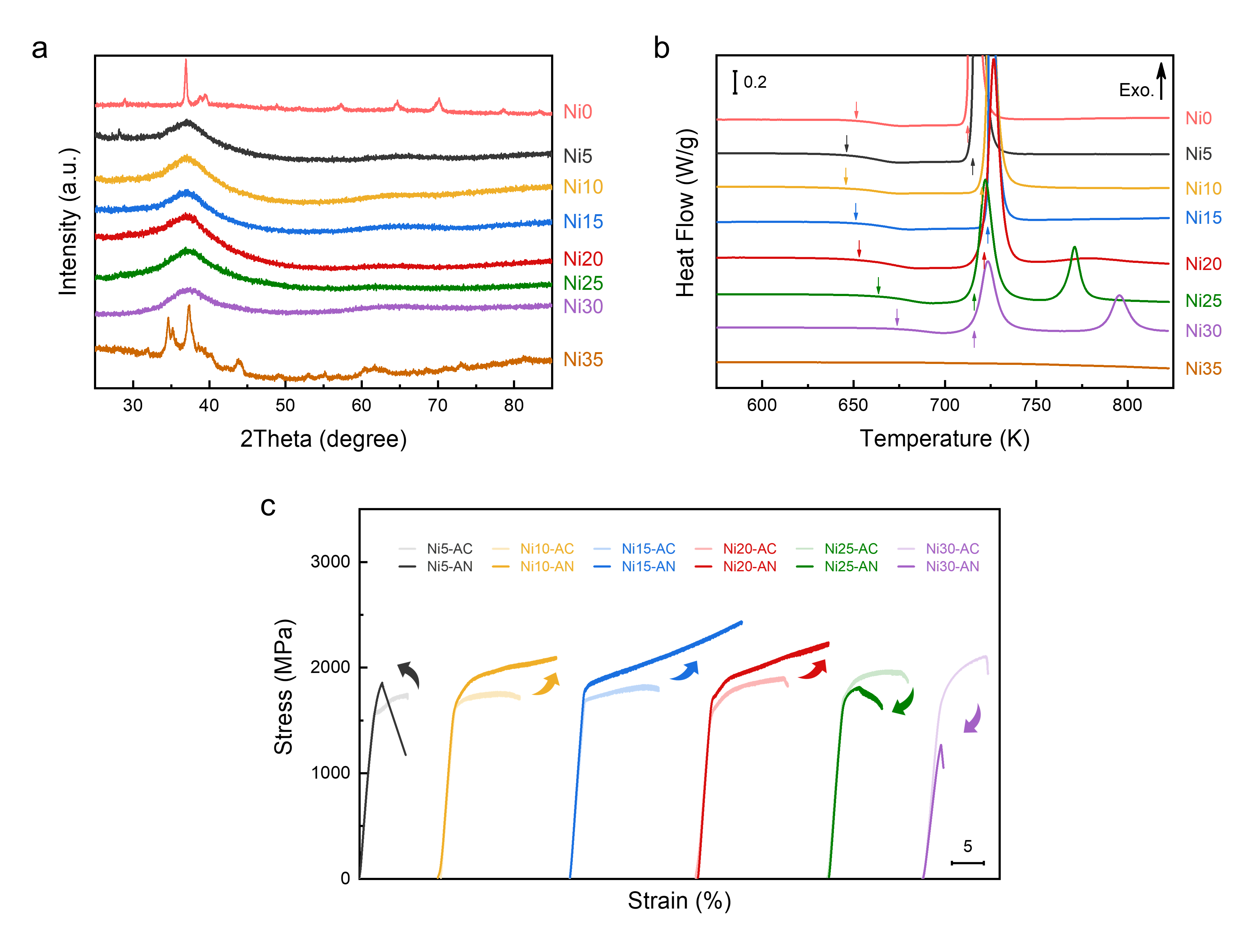
**

**Figure S18 Determination of optimal Ni content in Zr_60_Cu_35-x_Ni_x_Al_5_ alloys.** **(a)** X-ray diffraction patterns of Zr_60_Cu_35-x_Ni_x_Al_5_ (x = 0, 5, 10, 15, 20, 25, 30, 35) alloys. **(b)** DSC curves showing thermal stability and distinct crystallization events for each composition (heating rate: 10 K/min). **(c)** Compressive engineering stress-strain curves before and after optimal annealing, demonstrating the significant influence of Ni content on both strength and plasticity.

**References**

[1] T. W. Wu, F. Spaepen. "The relation between enbrittlement and structural relaxation of an amorphous metal." *Philosophical Magazine B: Physics of Condensed Matter* 61, no. 4 (1990): 739.

[2] P. Murali, U. Ramamurty. "Embrittlement of a bulk metallic glass due to sub- annealing." *Acta Materialia* 53, no. 5 (2005): 1467. https://doi.org/10.1016/j.actamat.2004.11.040.

[3] A. Slipenyuk, J. Eckert. "Correlation between enthalpy change and free volume reduction during structural relaxation of Zr55Cu30Al10Ni5 metallic glass." *Scripta Materialia* 50, no. 1 (2004): 39.

[4] J. Jackle. "Models of the glass transition." *Reports on Progress in Physics* 49, no. 2 (1986): 171.

[5] M. P. Moody, L. T. Stephenson, A. V. Ceguerra, S. P. Ringer. "Quantitative binomial distribution analyses of nanoscale like‐solute atom clustering and segregation in atom probe tomography data." *Microscopy Research and Technique* 71, no. 7 (2008): 542. https://doi.org/10.1002/jemt.20582.

[6] Y. Cao, M. Yang, Q. Du, F.-K. Chiang, Y. Zhang, S.-W. Chen, Y. Ke, H. Lou, F. Zhang, Y. Wu, H. Wang, S. Jiang, X. Zhang, Q. Zeng, X. Liu, Z. Lu. "Continuous polyamorphic transition in high-entropy metallic glass." *Nature Communications* 15, no. 1 (2024). https://doi.org/10.1038/s41467-024-51080-8.
